# ADAM: a robotic companion for enhanced quality of life in aging populations

**DOI:** 10.3389/fnbot.2024.1337608

**Published:** 2024-02-09

**Authors:** Alicia Mora, Adrian Prados, Alberto Mendez, Gonzalo Espinoza, Pavel Gonzalez, Blanca Lopez, Victor Muñoz, Luis Moreno, Santiago Garrido, Ramon Barber

**Affiliations:** RoboticsLab, Systems Engineering and Automation Department, Universidad Carlos III, Madrid, Spain

**Keywords:** elderly care, assistive robotics, physical assistance, social navigation, learning from demonstrations, environment perception, multi-robot tasks

## Abstract

One of the major problems of today's society is the rapid aging of its population. Life expectancy is increasing, but the quality of life is not. Faced with the growing number of people who require cognitive or physical assistance, new technological tools are emerging to help them. In this article, we present the ADAM robot, a new robot designed for domestic physical assistance. It mainly consists of a mobile base, two arms with grippers and vision systems. All this allows the performance of physical tasks that require navigation and manipulation of the environment. Among ADAM's features are its modularity, its adaptability to indoor environments and its versatility to function as an experimental platform and for service applications. In addition, it is designed to work respecting the user's personal space and is collaborative, so it can learn from experiences taught by them. We present the design of the robot as well as examples of use in domestic environments both alone and in collaboration with other domestic platforms, demonstrating its potential.

## 1 Introduction

Nowadays, we face a major global demographic challenge. United Nations analyses reveal a steady increase in life expectancy worldwide, having recovered and exceeded the values due to the COVID19 pandemic. Simultaneously, the birth rate continues to decline and this trend is expected to persist for the next 75 years (UN, 2023). This demographic dynamic translates into an unavoidable aging of the population, posing a far-reaching global challenge.

Focusing in the case of Spain, during 2021, life expectancy reached one of the highest levels in Europe, averaging 83.3 years. This statistic, supported by data provided by the “*Instituto Nacional de Estad–stica (INE),”* marked a slight recovery from pre-pandemic value (INE, 2023). However, it is important to note that, despite the upward trend in life expectancy observed before the pandemic, the age at which diagnosed chronic diseases begin to manifest themselves does not show a significant delay (Zueras and Rentería, 2021). This translates into a prolonged period in which people have to cope with these health conditions.

In this context, there is a pressing need to seek innovative solutions to ensure quality aging and provide adequate care for an ever-increasing elderly population, with new technologies emerging as a promising tool to significantly improve this situation (Ma et al., 2022). The application of robotics in this context emerges as a highly relevant contribution, by virtue of its ability to operate in human-inhabited environments without requiring substantial modifications. This approach has considerable advantages in the context of care and assistance to elderly individuals, allowing care to be provided in their home environment, and is supported by the opinion of healthcare professionals (Łukasik et al., 2020). A key element in the design of robots for this purpose lies in their versatility, which is manifested in their ability to carry out tasks of a domestic nature, such as setting the table, preparing food and cleaning the floor (Christoforou et al., 2020b). Their competence in the social sphere is also a significant virtue, insofar as these devices have the capacity to establish close interaction with the individual, thus mitigating the feeling of loneliness, and even allowing the monitoring of their state of health (Christoforou et al., 2020a).

Reviewing the attributes of the robots highlighted in these research studies, the RoboticsLab team from Universidad Carlos III de Madrid introduces the robot called *Autonomous Domestic Ambidextrous Manipulator* (ADAM), a mobile robot with bimanipulation capabilities designed specifically for the execution of domestic tasks, with the purpose of providing support to the elderly population in their homes.

**Figure 1:**
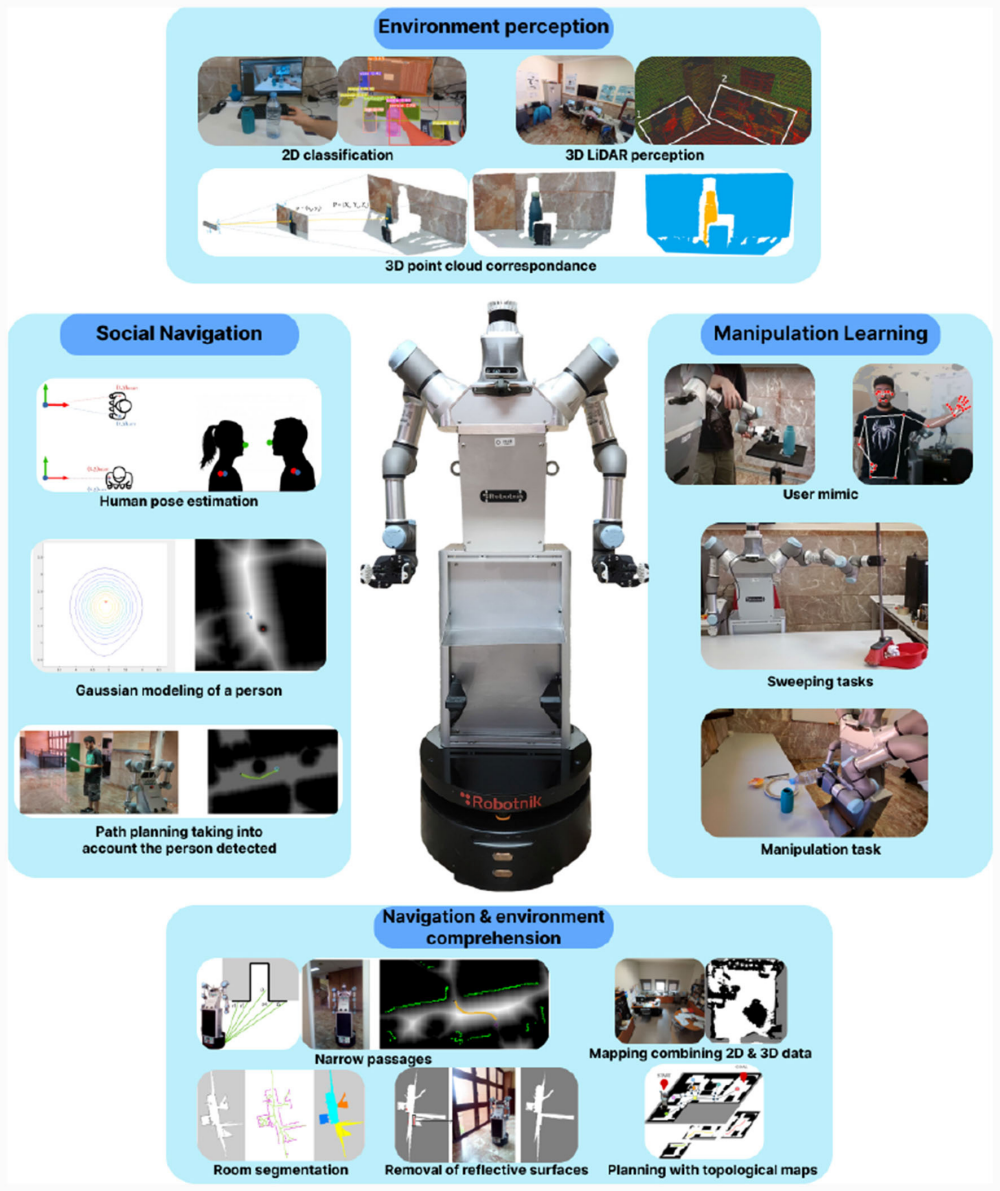
Visual description of the ADAM service robotic platform and its four main capabilities for the development of elderly care tasks: perception of the environment, navigation and environmental comprehension, social navigation and manipulation learning.

The ADAM robot is designed in collaboration with the manufacturer Robotnik. The configuration of the robot can be divided into four modules: perception system, mobile base, dual-arm system and robotic hands. The description of each of these components is detailed on Section 3.1. This configuration provide the robot with a number of particularly relevant capabilities to fulfill its function accurately and safely. These capabilities range from human awareness to the ability to comprehend the environment, as shown in Figure 1.

- Human awareness: by employing various sensors, the robot detects individuals in its surroundings, not only to prevent collisions but also to ensure it doesn't inconvenience people when carrying out specific tasks.
- Learning from the user: utilizing perception systems or through the collaborative capabilities of its arms, users can teach the robot to perform new tasks. For instance, they can instruct it in activities like floor cleaning.
- Detection and recognition: to execute tasks accurately, a perception system has been developed. This system can detect, recognize, locate, and even determine the shape of objects in the environment.
- Navigation and comprehension of complex scenarios: in pursuit of greater adaptability to its environment, the robot can comprehend the spatial context of the scenario. This allows it to differentiate between rooms and autonomously navigate in challenging situations, including negotiating doorways.

By making use of these capabilities, ADAM is able to provide quality care to the elderly people. The robot can carry out a wide range of household chores, from simple tasks such as picking up and delivering objects at the user's request, to more complex tasks such as cleaning the floor after learning from user's demonstration. In addition, it performs these tasks without modifying the environment, being able to provide assistance for these people in their own home. It should be noted that the maturity of these capabilities and their success will allow assistive robotics to play an important role in the future.

## 2 State of the art

Elderly care robots have been a main research topic in the last decades (Bardaro et al., 2022). These robots must be able to not only operate in unknown dynamic environments considering the humans with whom they share space, but also to be capable of interacting with all sorts of people by learning and adapting to them. These factors become even more relevant when working with elder people. When focusing on elderly care, it is very important to distinguish between two main approaches, cognitive care and physical task helper.

Cognitive care focuses on working on issues related to cognitive impairments and social interaction, such as the treatment of cognitive or psychotic disorders (Singh, 2022; Amaro et al., 2023). For this purpose, applications are developed in which these elderly people engage in stimulating interaction with the robot allowing them to work on issues related to memory or sensory perception (Andriella et al., 2020). Thus, robot for cognitive care design focuses on emotion recognition through visual sensors, speech recognition or even tactile sensors for the robot to respond accordingly, giving less relevance to the ability to manipulate the environment (Yamazaki, 2020).

Despite not being initially designed for elderly cognitive care, there exist several social robots which have been adapted to improve their adequacy to elderly care and companion applications. One of the most popular examples is the Pepper social robot developed by SoftBank Robotics (Japan), a commercial humanoid robot initially designed for business-to-business (B2B), later for business-to-customers (B2C) and business-to-academics (B2A) applications in stores and schools appealing customers and students thanks to its friendly appearance (Pandey and Gelin, 2018). Nowadays, Pepper is also used for and adapted to elderly care applications as the one presented in Takanokura et al. (2023), in which several elderly participants where brought together to complete cognitive tasks through interaction with the Pepper robot in a daycare facility. The NAO robot, a humanoid robot similar to Pepper but of smaller size, is another example of social robot which has been adapted to monitor vital signs in the elderly, such as blood pressure and heart rate. This is done by means of a sensor equipped platform as an extension to NAO named RIA (Vital et al., 2013). NAO was more recently used as part of a memory training program with mild cognitive impairment with participants between 45 and 85 years old (Pino et al., 2020), using visual recognition to detect emotions and gaze to assess the improvement in behavior after each session.

Other social robots are specifically built for elderly care applications from the start, guiding the design process with this purpose. This is the case of the Mini robot, designed for social companionship and domestic assistance, particularly catering to the needs of elderly individuals. This sophisticated robot employs a range of sensors and artificial intelligence to evaluate user engagement with tasks, enhancing interaction duration (Salichs et al., 2020; Mart́ınez et al., 2023). FRED is another robot specifically designed for elderly care which seeks to alleviate the symptoms of Alzheimer's disease and dementia through games and interactions with the robot (Mitchell et al., 2023). Even though cognitive assistance presents several psychological benefits as shown in the cited references, purely cognitive care robots fail to fully attend the needs of the elderly, and could be complemented with the advantages of physical assistance robots to help to perform demanding home tasks.

Physical task helper robots are responsible for assisting elderly people in the partial or total performance of everyday tasks which, due to their age or pathologies, they cannot perform optimally. These robots differ from those presented for cognitive care in that they must be able to interact with the environment that surrounds them. To do this, they must have actuators that allow them to manipulate different elements on the environment as well as sensors that help with said interaction. In addition to this, it is important to take the user into account at all times for these interactions. This allows to work safely in environments where both people and robots coexist.

Taking all these characteristics into account, there are a large number of robotic platforms which were designed in a generic way and were later given a utility aimed at helping older people (Asgharian et al., 2022). One of the most widely used general-purpose robotic platforms is TIAGo, an indoor mobile open-source robot for application of assistance that can achieve different tasks using its navigation, manipulation and perception elements. These robots have been used in projects like the presented in Muscar et al. (2022), where TIAGo's sensors are used to detect real time warning situations such as falls of elderly people in their homes and act actively in these situations. Another example of the use of TIAGo is the ENRICHME project presented in Coşar et al. (2020), where by using the robot's vision systems and the sensors of a home automation system, the robot is able to monitor and assist the elderly in any situation and help them with tasks by activating different elements of the home automation system. Another robot is ARMAR6 (Asfour et al., 2019), an initially designed collaborative humanoid primarily intended for industrial maintenance duties. It boasts essential functionalities like dual-arm mobile manipulation, accurate human pose estimation, and proficient object grasping capabilities.

Different models of assistive robots are also currently being developed. They are focused on interacting with actions and also helping in solving certain tasks that they are not capable to perform or that have limitations. An example of this is Gymmy, presented in Krakovski et al. (2021), which is a physical and cognitive aid robot that does not only assists the elderly in simple manipulation tasks, but also maintains and improves the independence of the elderly in the performance of certain tasks. For this purpose it uses a mobile base with manipulators and a screen that shows the elderly how to perform the task in case they need help. Another robot created specifically for assisting the elderly with household chores is the CHARMIE robot, presented in Ribeiro et al. (2021). This robot was tested during the COVID-19 pandemic and consists of a humanoid hand and manipulator, a mobile base with wheels and a head with a camera. CHARMIE was tested for tasks such as placing objects in inaccessible areas as well as for “carrying the shopping bag” tasks. One of the best known platforms is Hobbit, which from its inception was presented as a physical assistive robot for the elderly (Fischinger et al., 2016). A large number of works have been developed on this robot within this field, such as the one presented in Bajones et al. (2018), where it helps and prevents falls in elderly people. It also has the ability to pick up objects from the floor pointed out by the elderly, which can be potential sources of falls. This robot, like those previously presented, is formed by a manipulator arm, a mobile base and a camera to detect the elements of the environment. It also has a tablet with which the user can interact directly and give predefined orders. Finally, one of the most comprehensive current robots is GARMI (Tröbinger et al., 2021). This robot is made up of different modules that allow it to perform tasks such as manipulating elements of the environment, detecting objects and people and moving around in domestic environments. It also has a virtual reality-based support where the GARMI robot acts as an intermediary between the doctor and the user. To this end, they have developed a simulated environment where the user can connect with different members of the family or the doctor via video calls, and they have even set up certain cognitive rehabilitation exercises for users.

Following this classification, the ADAM robot presented in this work has been created specifically as a robot to physically assist elderly people and also as a platform for research and development of new techniques for performing tasks in indoor environments as efficiently and safely as possible. ADAM, being an indoor robot and working in domestic environments, has a series of characteristics in common with the robots previously presented, such as adapting its size to homes to allow it to work safely, passing through doors and being able to manipulate the different elements of the environment (such as objects or furniture). Despite this, ADAM presents certain elements that differentiate it from the rest of the robots described in this section:

- ADAM is formed by a combination of modular systems, facilitating seamless integration of multiple sensory inputs from the cameras, arms, hands and base. Each component within this system can work both independently as well as being able to work in a coordinated manner. Additionally, each module allows to work in low and high-level, turning ADAM into a suitable platform for research as well as for elderly user care tasks.
- People in the environment are taken into account at all times when carrying out tasks. This makes it possible to work safely on indoor environments, generating collision-free movements with users.
- The robot arms are collaborative. This allows users to operate them and adapt their movements according to the characteristics of their own environment in each case. It also has a series of safety criteria where, if it detects collisions, it stops to avoid any type of damage to the environment or to the elderly person.

## 3 Robot design and integration

The ADAM robot is a dual-arm mobile manipulator robot with the capability to perform various assistive household tasks. In order to be able to perform these tasks, the robot has been designed to adapt to a human environment, so its dimensions are in accordance with this. Also its cognitive capabilities must be appropriate to facilitate the coexistence of the platform with the users. In this section, a detailed description of the physical and software design of the robot is given.

### 3.1 Robot components

**Figure 2:**
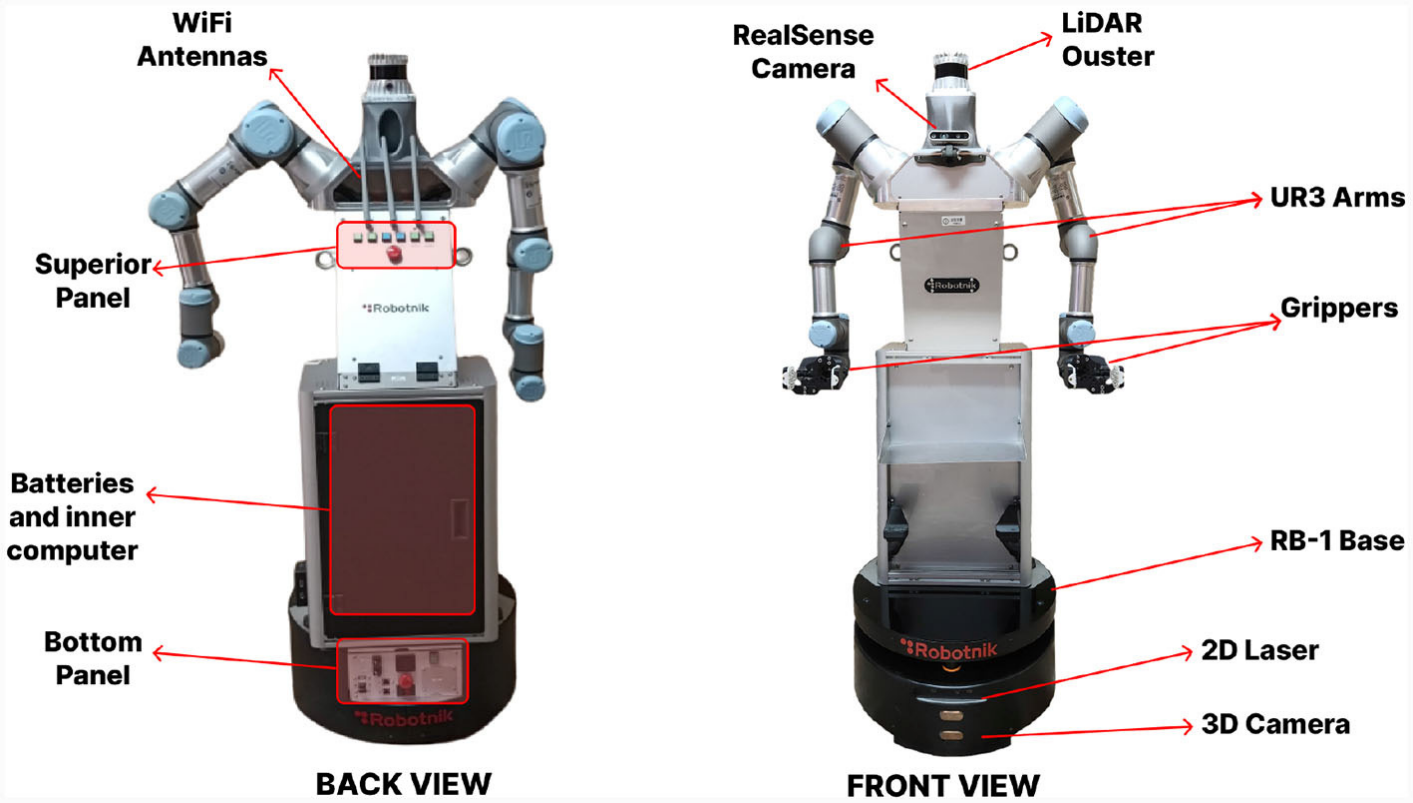
General scheme of ADAM elements from back and front view.

The robotic platform is composed of several modules that enable the performance of navigation and manipulation tasks for the execution of physical assistance tasks. It comprises a perception system, a mobile base, a torso, two arms and two grippers, reaching a total height of 160 cm and a 50 cm width when the arms are at rest. These systems are independent and commercial modules that have been connected together to form the total robot structure. A schematic of these elements can be seen in Figure 2.

The robot is completely modular, with its different parts being able to work individually as well as together. In addition, the robot is completely autonomous, having batteries in its base that provide it with enough energy to move both the base and the arms and to include additional sensors such as cameras or 3D LiDAR sensors. The average battery life is derived from the number of simultaneous active modules, with the maximum battery life achieved solely with the base module (using only the navigation module) estimated at 9.3 h. The minimum battery life, with all modules connected and functioning simultaneously, is 3.8 h. The estimated approximate charging time for the batteries is 2.2 h. ADAM also has two central computers, one for the base and one for the arms, which have the controllers to act on them. These two computers are connected via an internal cable network. In addition, the robot has a WiFi module that allows the robot to communicate with external computers where we execute custom made algorithms that process sensor data and send commands. Each of the different modules of the robot, both its own (base and arms) and those added by us (perception system and manipulators), are presented in detail below.

#### 3.1.1 Perception system

The perception system allows to capture relevant information from the environment to perform navigation and manipulation tasks. The robot has sensors that are already integrated into its commercial components. The robotic base includes an RGBD camera and a 2D LiDAR. These sensors are located practically at ground level, a few centimeters above the ground, and pointing toward the front side of the robot. This means that their range of vision is quite limited with respect to height. As mentioned in Section 3.2.2, they are useful for low-level actions like measuring distances at specific times. However, it is necessary to include additional devices that extend the range of vision and allow higher-level tasks to be performed. The proposed selection of additional components is detailed below.

The additional sensor selection for the perception system on the ADAM robot combines depth sensors with RGB cameras with the main purpose of detecting and localizing elements of the environment in space, which is essential for both manipulation and navigation tasks. The core components of this system mainly comprise two sensors: the Realsense D435 depth camera and the Ouster OS0 LiDAR sensor. Their seamless integration into the Robot Operating System (ROS) facilitates communication with other robot components. These sensors have a preinstalled software that can be used in some simple robotics applications, but to perform more complex tasks, typical of an assistive robot, we develop our own software that in certain cases uses some preinstalled utilities. In addition, due to their versatile characteristics, these sensors find applications in a variety of tasks.

**Figure 3:**
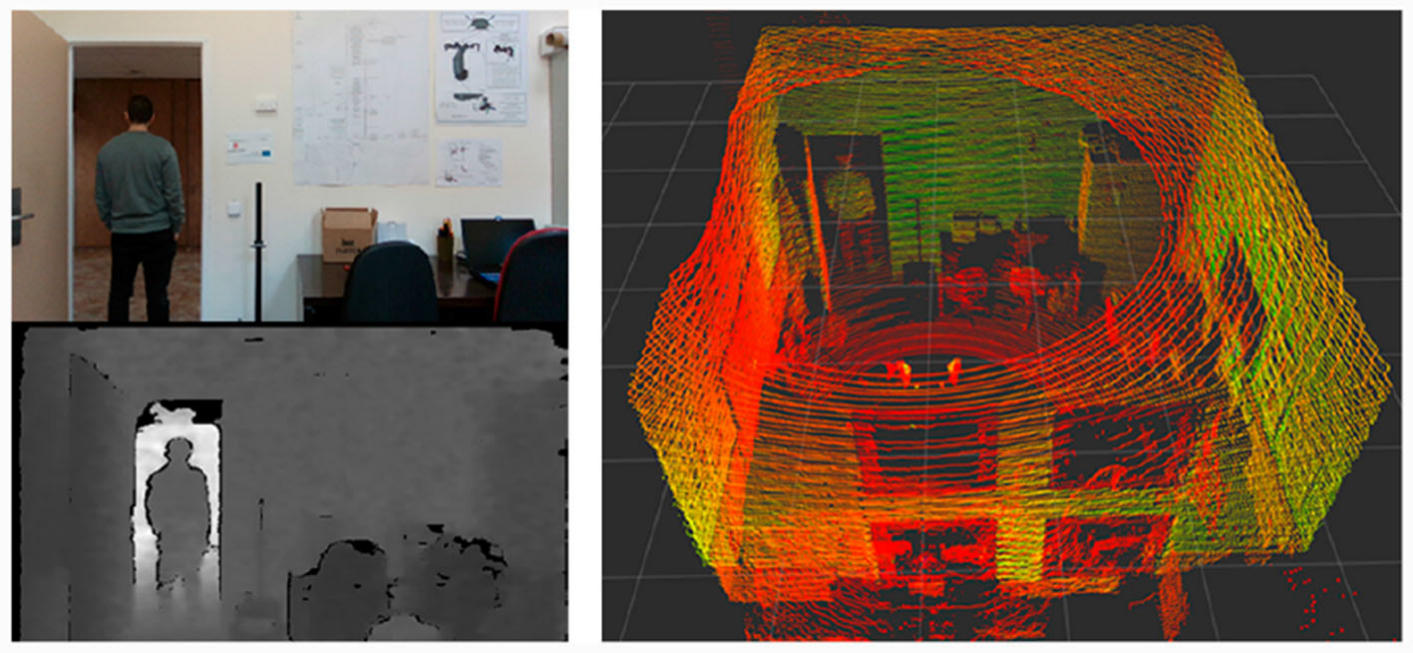
Information captured by the perception system. The main sources of information are the RGB image and the corresponding depth values from the RGBD sensor and the 3D spatial information from the LiDAR sensor, which covers a full room.

The Realsense D435 depth camera is an RGBD sensor, consisting of an infrared stereo vision and a traditional RGB module. In terms of specifications proper to this type of sensor, this camera has a maximum resolution of 1,280 × 720 for the depth stream and 1,920 × 1,080 for the RGB stream, with a frame rate of 90 and 30 fps respectively and a field of view of 87° × 58°, with an operating range of up to 3 m, an example of the output given by these two streams can be seen on Figure 3. This specifications allow to obtain this information accurately with a precision error of < 2% at 2 m. This sensor is placed on a mobile support that allows to modify the camera angle and its position depending on the task to be performed, so its coordinate transform is variable.

The LiDAR sensor Ouster OS0 allows to obtain a greater range of perception both in angle and range. Specifically, it has a maximum range of 100 m and a vertical field of view of 90° and 128 channels of resolution. With this specifications it can obtain information from almost all the entire room in a single sweep of the sensor, as shown in Figure 3, if there are no occlusions that may generate shaded areas affecting the final map result. It is important to note that it does not only provide spatial information, but it can also detect other properties such as reflectivity due to the nature of the sensor. By installing the sensor on top of the robot, occlusions with its own body are avoided, maximizing its capabilities. The coordinate transformation with respect to the robot is given by a translation from the LiDAR to the base. This corresponds to 130 cm in the *Z* axis.

These sensors have been applied to develop a system for detecting and recognizing the environment, obtaining both 2D and 3D information. This information gives the robot the ability to perform manipulation and navigation tasks accurately and safely. The detailed description of these applications is developed in Section 3.2.1.

#### 3.1.2 Mobile base

The navigation capabilities of the robot are determined by its mobile platform, specifically the RB-1 model manufactured by the company Robotnik. This device has dimensions of 50 cm in diameter and it is equipped with two motorized wheels and three supporting wheels. This configuration enables the robot to move both forward and backward, as well as rotate in place, facilitating the seamless combination of these two movements. It should be noted that lateral displacement is not within the capabilities of this base.

As mentioned above, the base has two integrated sensors, an RGBD camera and a 2D laser. The base is designed as a stand-alone module, so in its original design these sensors are sufficient for the classical navigation algorithms that this base can perform. However, in our case, we have additional elements mounted on it, more specifically a torso and two industrial arms, so both perception and navigation strategies need to be adapted. We have developed customized algorithms to consider the facts when navigating as explained in Section 3.2.2.

#### 3.1.3 Dual-arm system

The design and selection of the ADAM robot manipulators is governed by two main characteristics. The first is its body composition. The robot has to meet physical standards that simulate the structure of a human torso and arms. This is because a human-like structure allows it to work more comfortably in domestic environments because the rooms, doors and furniture are adapted to humans. The second feature is specific to our own design, and that is that we want the arms to be collaborative. These arms allow you to reprogramme and reallocate movements as needed in all your operations, maximizing flexibility, efficiency and productivity. They are also equipped with safety systems to keep the elderly safe from collisions when performing joint tasks. With these two characteristics in mind, it was decided to use the UR3 models provided by Universal Robots.

**Figure 4:**
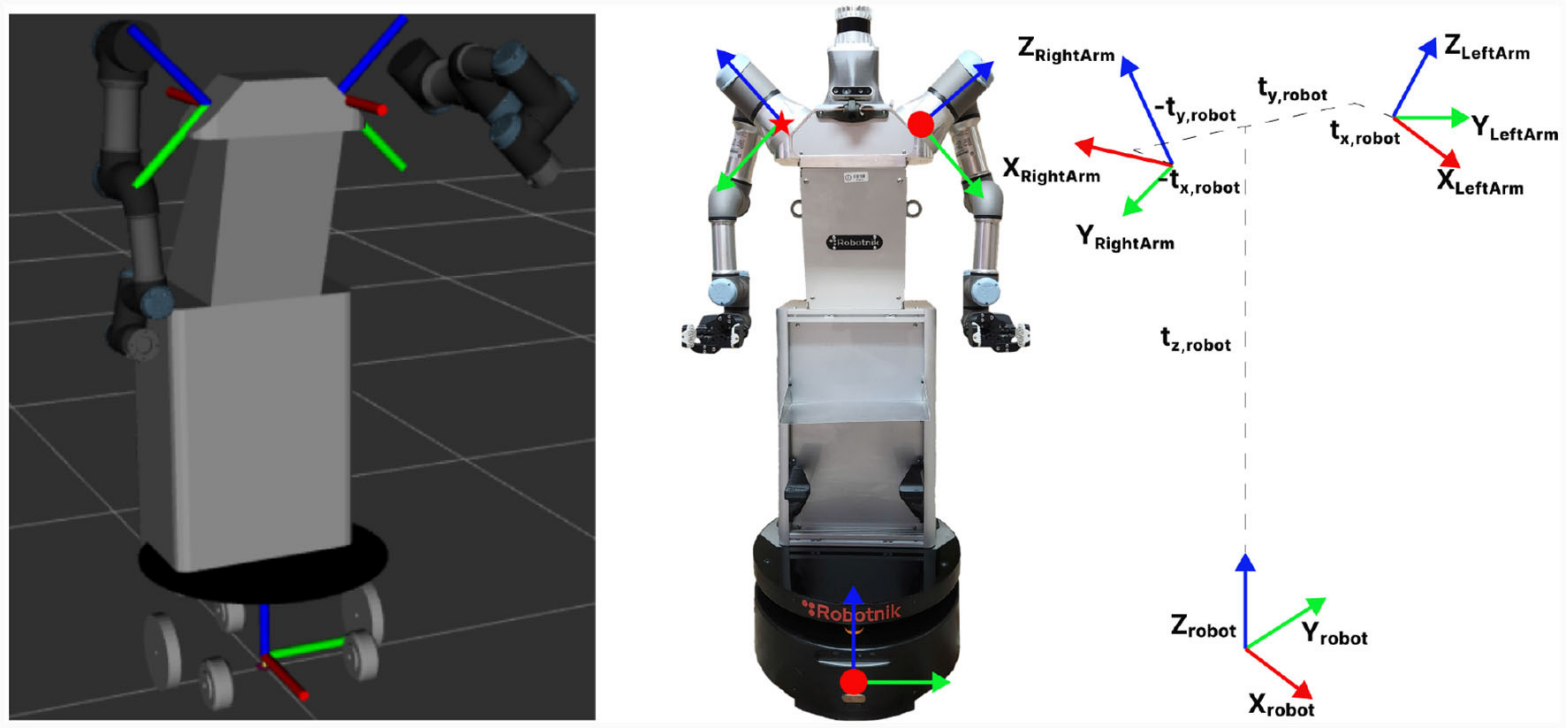
Visualization of the ADAM model in simulation, where the reference systems of the base and arms can be seen. The reference frame transformations between them are schematically represented.

These arms have a total length of 50 cm and a maximum load capacity of 3 kg. Each arm is made up of 6 degrees of freedom (DoF) with a range of movement between ±360°, with the exception of the end effector which allows more than one turn. To achieve human configurations with the UR3 arms, which are non-anthropomorphic, the positioning of the arms has been reconfigured. The arms have been rotated with respect to the robot base (understood as the global reference of the system) 45° for both arms. In addition to this, the arms have been positioned on the torso of the robot by means of a displacement in the three *XYZ* axes with respect to the base. To establish the connection between these three reference axes, it will be essential to perform both rotational and translational operations. This will enable us to ensure that all the points computed concerning the robot base by the sensors can subsequently be associated with the arms, preventing any errors in movement execution. This procedure relies on a sequence of transformations in which the joint positions are transmitted to create the necessary trajectory in the physical model of the robot. This process is expressed for the left arm as *H*_*left*_ = *T*(*t*_*x, robot*_, *t*_*y, robot*_, *t*_*z, robot*_)*T*(*x*, −π/4) and for the right arm as *H*_*right*_ = *T*(−*t*_*x, robot*_, −*t*_*y, robot*_, *t*_*z, robot*_)*T*(*z*, π)*T*(*x*, −π/4) as is presented in Figure 4. These translations make it possible to reference the handling system with respect to the overall robot system, which facilitates the processing of the information to work together on the whole system.

For manipulation we make extensive use of the collaborative capabilities of the UR3 arms. First of all we exploit the use of *gravitational compensation*. This mode allows us to move the arm directly by hand without the need of teleoperation, that allows us to capture data with it in a very simple and intuitive way. These capabilities are exploited in performing tasks that are adapted to different human environments. Additionally, if the arm detects a collision with an external elements it blocks and enter in a safety mode, that uses the gravitational compensation. The presented capabilities allow them to work safely in human interaction. In our case, these capabilities focus on learning tasks by imitating the human user. UR3 arms has the ability to be programmed using code by an expert or to be moved by a user. This capability of programming just with the movement allows us to specify, in a very simple way, tasks that through classical programming could be very tedious to adapt to highly changeable environments such as homes. The application and exploration of these capabilities is developed in depth in Section 3.2.3

#### 3.1.4 Robotic hands

**Figure 5:**
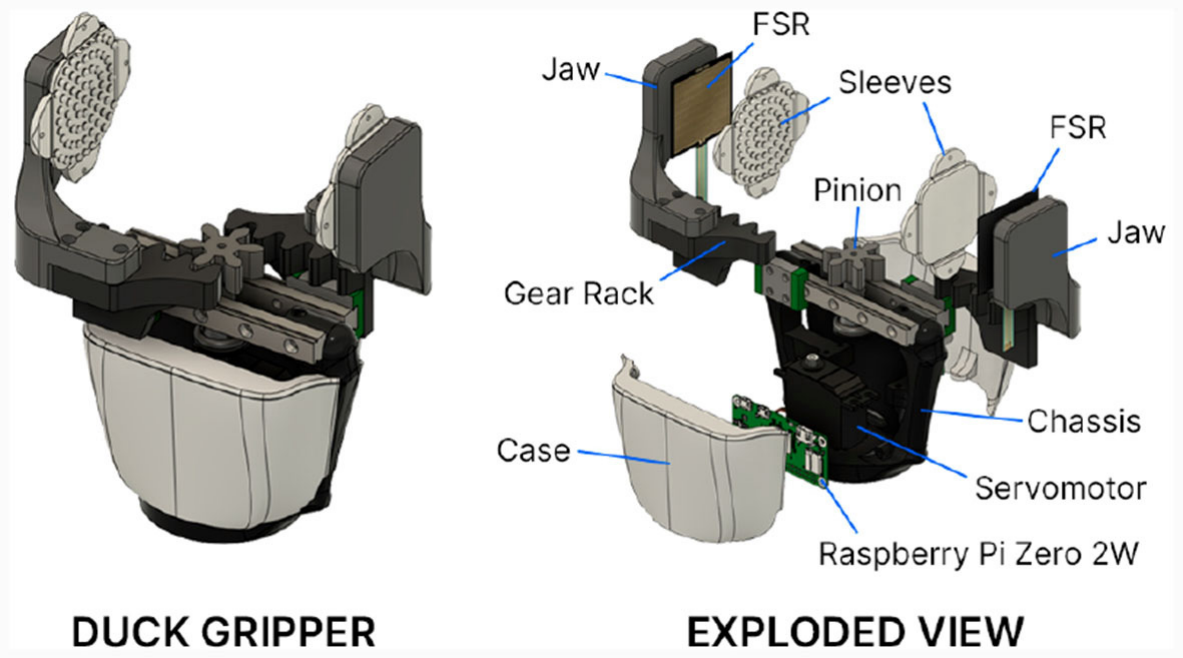
Duck Gripper final design with an exploded view of the gripper and its main components.

To grant complete manipulation capabilities to the robot and allow it to grasp everyday objects, the parallel-jaw gripper named *Duck Gripper* was designed (Figure 5). The device constitutes a simple and ready to use end effector as a physically independent module to be easily mounted on the robot arms so it does not require any further wiring, but which can be controlled by the robot's central system via ROS over Wi-Fi communication. To achieve this, the gripper is equipped with its own power supply and a Raspberry Pi Zero 2 W board with an integrated Wi-Fi module to execute and communicate the ROS node associated to it. This gripper node is responsible for reading measurements from two Force Sensing Resistors (FSR's) mounted on each jaw to actuate the servomotor that drives the mechanism based on the obtained information.

The gripper has a total height of 180 and 118 mm width measured on its body, and total width of 148 mm measured on the jaw outer faces when the gripper is completely open. These dimensions prevent self-collision between the gripper and the arm. As shown in the exploded view in Figure 5, the chassis acts as the main skeleton of the gripper supporting the electronic components, the cases that protect them and the sliders for the gear racks. Regarding the mechanism, a rack and pinion pair is used to transform the rotating motion of the actuator into linear motion to each jaw. This mechanism sets a maximum amplitude of 117 mm between jaws and minimum of 35 mm (a stroke per jaw of 41 mm). The actuator is the FEETECH high-torque servomotor FS5115M-FB with position feedback, directly connected to the Raspberry Pi. This servomotor rotates from its initial position closing the gripper until the object to be grabbed is detected by the FSR, and it stops when the force sensed exceeds a force threshold on each FSR.

The gripper actuation is programmed in a ROS node (*/LeftGripper* or */RightGripper*) which publishes a change in state between “object grabbed” and “object dropped” which is set by the force threshold. The command to open and close is received by the gripper as a boolean message via a ROS topic to which the gripper node is subscribed, and the gripper blocks its motion whenever the connection to the robot is lost. The communication between the gripper and the robot is further explained in Section 3.2.4.

**Figure 6:**
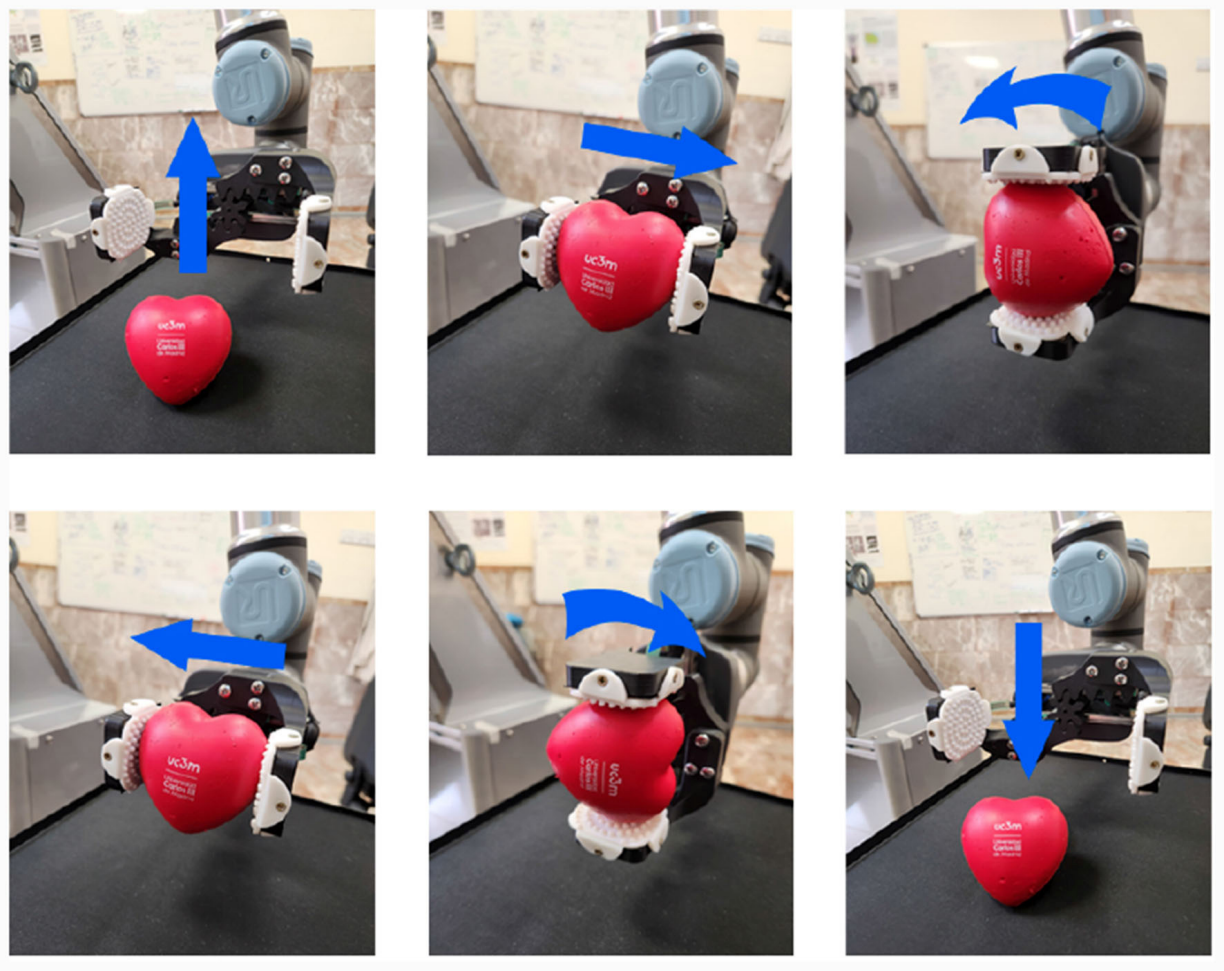
Duck Gripper performance test movements. From left to right, and from top to bottom. The gripper grabs the object on the workstation. The gripper displaces away from the robot. The end effector rotates 90° clockwise. The gripper displaces toward the robot. The end effector rotates 90° counterclockwise. The gripper opens to release the object.

The performance of the gripper is tested on various everyday objects of different sizes, weights and mechanical properties, such as bottles, boxes, fruits, and anti-stress balls. On each test, the gripper performs a series of six movements which simulate a task in which an object is grabbed and displaced as in real world applications (Figure 6), considering a successful grab when the object is not dropped nor damaged throughout the entire test and when the correct ROS messages are sent. Various thresholds are selected increasing the force required to grab, and 10 tests are performed for each object and threshold obtaining the results in Table 1. The value with highest grab success rate across all objects is selected as the standard configuration of the gripper.

**Table 1:** Grab success per object type and weight for different force thresholds.

**Objects**	**Weight (g)**	**Threshold (V)**						
		**0.25**	**0.5**	**1**	**1.5**	**2**	**2.5**	**3**
Anti stress ball	20	10	8	8	10	10	10	8
Uncooked egg	57	10	10	10	10	10	10	10
Rigif 3D printed bottle	100	10	10	10	9	10	10	10
Apple	157	10	10	10	10	10	10	10
Half-full 1L water bottle	500	2	4	10	10	10	10	2
Cardboard box	815	1	3	10	10	10	2	0
Filled 1L water bottle	1.025	0	0	0	4	10	10	0
Glass jar with water	1.500	0	0	0	0	0	0	0

In the final gripper configuration, the gripper has a total weight of 0.49 kg, it has a grab capacity of objects up to 1.20 kg without dropping the object, and its battery life ranges from 2 to 9 h depending on use (4 h recharge time). These specifications are more than adequate for its final use together with the robot, which is further shown in Section 4.1. Two identical models of the *Duck Gripper* are developed and mounted on the left and right robot arms.

### 3.2 Functioning modules

The robot, as mentioned above, is modular, so each of its parts can work independently. Each of the physical modules has a software module assigned to it that characterizes its operation. These modules have to be connected to each other for their coordinated operation. In addition, the robot can be connected with other robotic devices to work together. A detailed description of these features is given in this section.

#### 3.2.1 Vision software

In order to perform any task in an environment, obtaining feedback is imperative. Humans, for example, use their five senses: sight, hearing, smell, taste, and touch, to perceive the world around them. When it comes to physically interacting with the environment, we tend to be guided primarily by the senses of sight and touch. Following this inspiration, in order to be able to physically interact with its environment and successfully carry out manipulation and navigation tasks, a robot needs to be equipped with a perception system analogous to the sense of sight.

We have developed different algorithms to provide the perception system the ability to obtain visual information from the environment. In the case of the RGBD camera, we have focused mainly on three key developments: the detection and tracking of relevant human body points, the detection and localization of daily objects, as well as the extraction of point clouds of objects of interest. For the LiDAR sensor, our algorithms focus in the construction of high-level maps and the detection of reflective surfaces.

**Figure 7:**
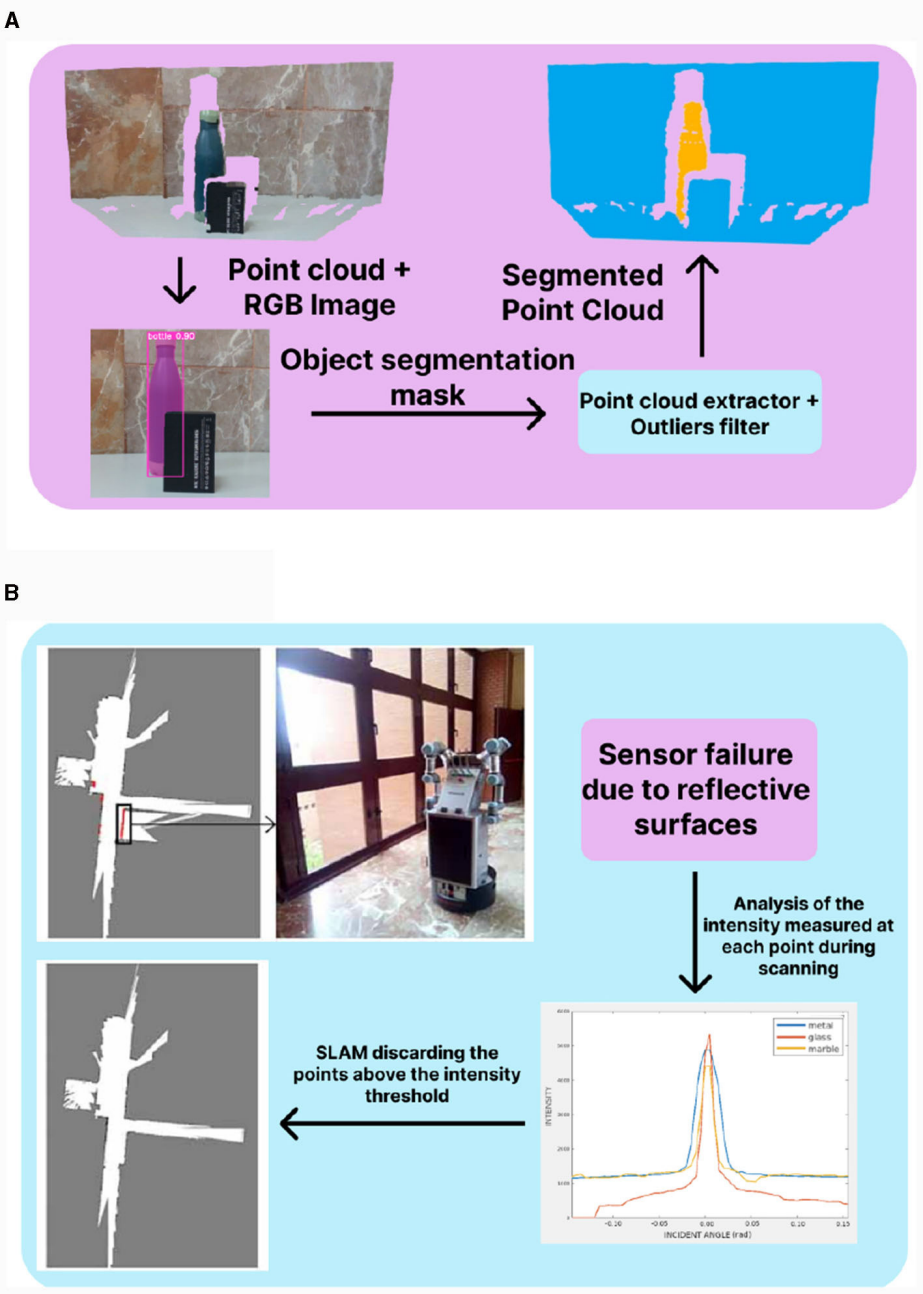
**(A)** Example of Point Cloud Segmentation presented in Mora et al. (2023b). Using the RGBD camera, the robot take a RGB image and a point cloud as input. Process the image with YOLO, then extract the mask of the object. Finally, projects the mask onto the point cloud and filter outliers, obtaining the segmented point cloud of the object. **(B)** Example of Detection of reflective surfaces based on intensity presented in Mora et al. (2023c). In this case, the robot takes the input from the LiDAR sensor, but in this situation the measurements fails, making an analysis of the intensity values for each points during mapping, these errors can be solved. To do that, in the analysis of the intensity values a threshold is calculated. With this threshold into the SLAM algorithm, all the points over are discarded, obtaining a final map without noise.

Our proposal for the detection and tracking of relevant human body points is made using the Google's algorithm Mediapipe presented in Lugaresi et al. (2019) and Zhang et al. (2020). This algorithm tracks several points of the human body in pixel coordinates, then making the pixel correspondence to the depth frame captured by the RGBD camera, the pose information of the human body points can be obtained. With this information the robot is able to perform tasks such as social navigation and learning from user, explained in detail on Sections 3.2.2 and 3.2.3 respectively. To perform the detection and localization of daily objects, our solution combines information from the two data streams of the RGBD camera, that is, RGB and depth data. This is done by using Deep Learning techniques involving the detection and segmentation of objects in the RGB image. For that purpose, we use You Only Look Once (YOLO) Redmon et al. (2016), a state-of-the-art, real-time object detection algorithm, whose main advantage is that it uses features from the entire image to predict each bounding box and is able to make all the predictions simultaneously, allowing an object detection with high speed and average precision. In this case, we use a recent version that performs instance segmentation, specifically the Ultralytics's YOLOv5x-seg network that has a mean average precision in masks estimation (*mAP*^*mask*^) of 41.4% in COCO dataset (Ultralytics, 2022). This segmentation provides a mask for each detected object, that is used to extract its center point, calculating its centroid with classical computer vision techniques. Then, with depth information, the distance between the object center and the camera is determined. Finally, by applying coordinate transformations, the actual position of the detected objects in relation to the robot base is obtained. Similarly, we developed another algorithm that is used to extract point clouds of the detected objects, which allows for a more precise localization. Both processes start from the same detection in the RGB image, using the same network. But in this case, the segmentation points provided by the instance segmentation are filtered out and extracted from the point cloud resulting from the stereoscopic vision of the sensor, which captures the real three-dimensional shape of the object. This process can be seen in Figure 7A. To state that this technique is the best for an accurate 3D object localization, we performed some tests with different objects (smaller objects in workspaces like bottles and larger objects like chairs or sofas) and the following methods: region growing (RG), LCCP, grab cut (GC) and instance segmentation (INST). In Table 2 there is a summary of the results obtained using various metrics. It was obtained that all methods have better results with workspace objects, except Instance Segmentation that provides the best results in both and in almost all metrics. This research was presented in the paper (Mora et al., 2023b) where we discuss all these techniques in detail.

**Table 2:** Performance metrics for the four proposed methods.

	**Time (s)**	**IoU**	**CD (m)**	**Distance (m)**	
RG	0.0599 ± 0.0105	0.5808 ± 0.2623	4.9550 ± 10.2449	0.0327 ± 0.0426	Workspace
	0.4415 ± 0.2291	0.4512 ± 0.2243	997.7513 ± 841.9354	0.2085 ± 0.1225	Larger
LCCP	**0.0058** **±0.0044**	**0.6042** **±0.3363**	7.7770 ± 10.8200	0.0489 ± 0.0872	Workspace
	0.0383 ± 0.0217	0.4551 ± 0.2591	704.7949 ± 522.7520	0.2250 ± 0.1773	Larger
GC	1.0789 ± 0.1589	0.5076 ± 0.2330	5.0067 ± 2.8707	0.0326 ± 0.0373	Workspace
	2.3043 ± 1.3372	0.4525 ± 0.2566	600.1952 ± 490.6789	**0.1934** **±0.1631**	Larger
INST	0.0070 ± 0.0027	0.5246 ± 0.2391	**4.4195** **±3.5965**	**0.0263** **±0.0302**	Workspace
	**0.0124** **±0.0083**	**0.4763** **±0.2038**	**459.2447** **±425.6846**	0.2195 ± 0.1367	Larger

Bold numbers indicate which method obtained the best values for the proposed metrics.

For the construction of high-level maps we have develop an algorithm that uses the 3D information given by the LiDAR sensor. This valuable information makes possible to better identify the occupied an unoccupied areas, even in complex situations for mapping algorithms such as chairs or tables. Section 3.2.2 provides a detailed description of this process. Although the information given by the LiDAR sensor is highly relevant for mapping algorithms, it can also introduce measurement errors. This failure happens when the surface material is reflective, since projected rays may not even strike the sensor back. To prevent these errors on the final map, the solution consist of the detection of the intensity peaks and filter those points that exceed a threshold, as shown in Figure 7B. To extract the value of this threshold, we make several tests with different incident angle, lighting conditions and distance for various surface materials. In this work, glass, metallic and marble surfaces are considered as reflective. Painted walls are treated as non-reflective surface. During the test, we observe that there as gap on the intensity measurement between reflective and non-reflective surfaces. The peaks of this measurements are collected in Table 3. From this results, it is obtained that the threshold must be over 2,000 units, because all reflective surfaces are above this value, while painted wall intensity peaks are kept below this value. This solution was presented in Mora et al. (2023c) and is used as filtering step in other mappings algorithm to get better results.

**Table 3:** Intensity peaks measured on reflective and non-reflective surfaces.

	**Glass**	**Metal**	**Marble**	**Painted wall**
Min peak	6.502	3.760	2.864	1.173
Max peak	8.749	4.987	4.472	1.681

#### 3.2.2 Environment mapping and navigation

Just like people, robots need to know where they are in order to be able to perform activities in the environment and interact with it. In an initial stage, when accessing an unfamiliar environment, people capture information from the environment so that it is familiar to them thereafter. This is done by robots as well and is known as mapping. The result of this task is a map with relevant information of the environment. In our case, it is important that this representation is robust and secure for applications to be user friendly. In addition, it must have several levels of information, from the lowest to the highest, to facilitate communication with people.

ADAM features a low-level geometric mapping algorithm based on the Ouster 3D sensor. From the 3D spatial information, we propose the construction of a robust map in which the complete geometry of the objects is considered. Thanks to the position of the sensor (on top of the robot), objects such as tables can be mapped in their entirety, not just a part as might be the legs, so the model is more faithful to reality. In addition, reflectivity information is integrated, so surfaces such as glass or metal walls are detected and filtered. For this purpose, an algorithm has been developed in which 3D spatial information from the laser scan sensor and intensity information is mathematically analyzed and used to define a sensor profile, where information is projected onto the 2D floor plane. A sensor profile is extracted for every 3D scan that is captured, so for every data capturing step, there is a corresponding 2D sensor profile. Then, 2D data is merged using a recursive Bayesian filter modeled as a Markov Random Field of order 0 to create the final map, meaning that each cell in the map is estimated as an independent variable (Mora et al., 2023a).

**Figure 8:**
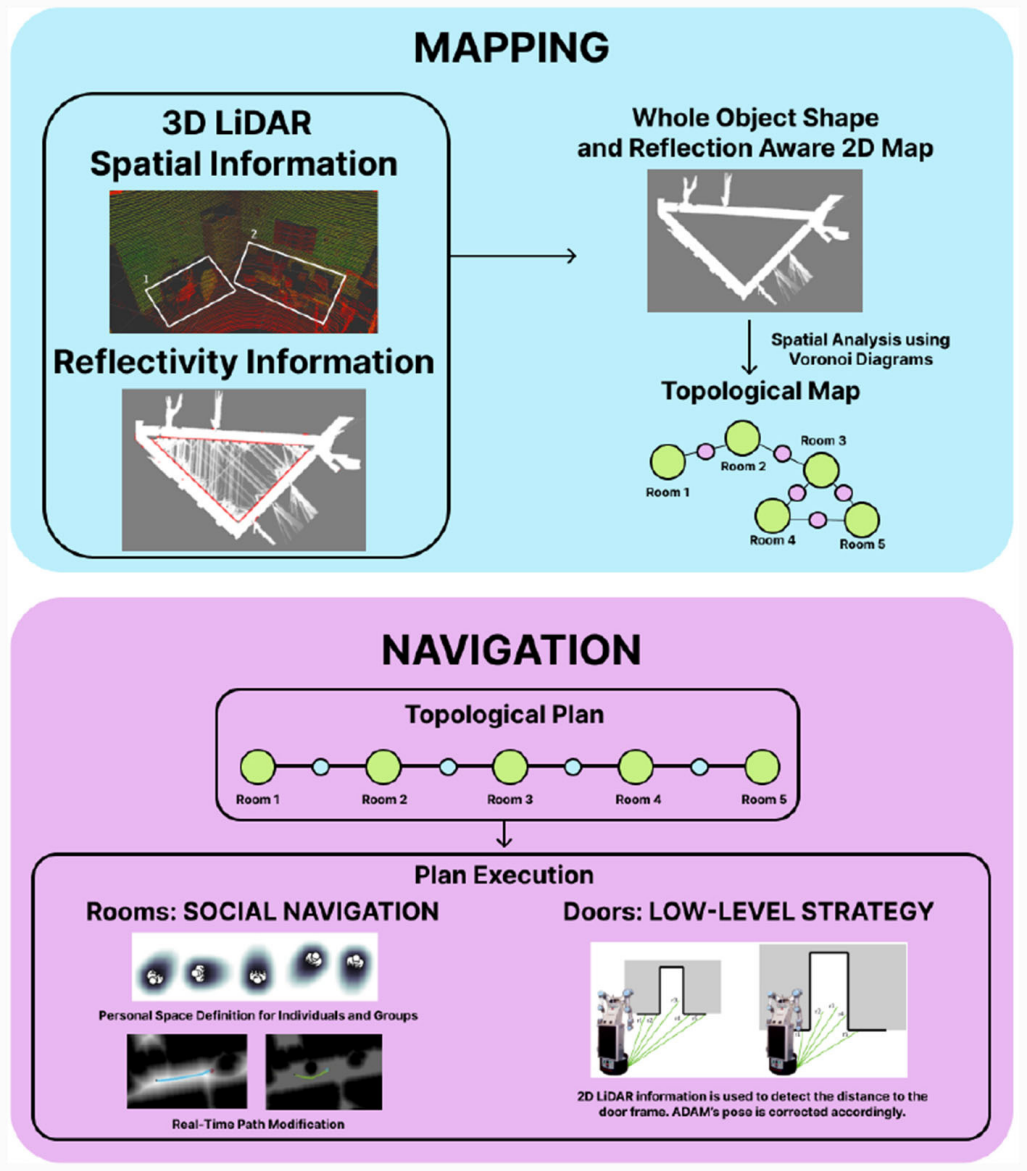
ADAM's mapping and navigation capabilities. The 3D LiDAR is used to obtain spatial and reflectivity information, turning into a 2D geometric map with whole object shape and reflectivity awareness. Voronoi diagrams are applied to extract the topological structure of the environment. With that information, a topological path is planned, containing a sequence of rooms (green) and doors (blue) to be traversed. Social navigation is performed in rooms, avoiding people's personal space. Doors are traversed using a low-level strategy using the 2D LiDAR information.

However, geometric maps are difficult for people to interpret. When a robot receives a command from a user, it is not in geometric coordinates. The command is usually related to a room in which to perform a task, such as “go to the kitchen” or “go to the bedroom,” Therefore, a map closer to the way people partition their environment is needed. By applying Voronoi diagrams to geometric maps, the environment is partitioned into rooms, creating a topological map in which the environment is identified with a graph. Voronoi diagrams partition free space into regions based on proximity. By looking for the regions of the diagram closest to occupied areas, it is possible to identify narrow passages. Other works have already proposed to use these diagrams for partitioning the environment. Our novelty is applying Voronoi diagrams not only in free space but also in occupied space. While diagrams from free space indicate where narrow zones are found, those extracted from occupied spaces indicate protruding areas. By combining both approaches, narrow passages are effectively found (Gonzalez et al., 2022; Mora et al., 2022a). Once rooms are clearly differentiated, they can be labeled according to the observed objects inside it. By using object-room co-occurrences, which indicate how probable it is to find a certain object inside a room, topological rooms are labeled into room types. A summary on how the proposed maps are constructed and used is shown in Figure 8.

Once the maps are available, the robot uses these representations to navigate the environment. Given the social nature of the robot's applications, where it must assist users, it is important to ensure safety during movement. One of the most representative algorithms of the ADAM robot that we have developed is *social navigation*. This algorithm has been designed to not only prevent the robot from colliding with dynamic elements but also to respect the users' personal space. In this way, the robot is not only safe but also comfortable to use. For this application, the topological map is first used to calculate the sequence of rooms and doors that the robot will have to pass through. Once calculated, two different navigation strategies are used depending on whether the robot must traverse a narrow passage or navigate a wide area.

In the first case, the robot should follow a straight line to cross a passage, but due to irregularities in scenarios such as floor bumps, it robot tends to leave path. Then, the 2D LiDAR on the mobile base is used to adjust its pose with greater precision. This is essential, as the diameter of ADAM's base is of considerable size, leaving little clearance between the robot and the door frame, increasing the risk of collision. If the measured distance is smaller on one of the robot sides, an angular velocity is added with opposite direction until the error is compensated. The video https://youtu.be/BAvnfvSnMmo presents the operation of the algorithm generated for traversing narrow passages.

In the second case, the robot navigates over a wider area. The path that must be followed in a room is calculated by Fast Marching Square. This method is based on the way in which light is propagated in space, which applied to an occupancy grid map results into a matrix where each cell indicates the arrival time of the wave. This matrix is also known as *velocity map*, and it will serve as an indication of how fast the robot can move on each part of the map. This algorithm is capable of finding the shortest path on the velocity map while optimizing speed, that is to say, time. Some major highlights of this method are the capability of finding the fastest possible path, being smooth and avoiding local minima. In addition, if an obstacle that was not previously included in the map is encountered by the perception system, it is included and the path is recalculated. As a social component, the robot is able to detect the pose of the people around it. To do so, the skeleton detection method MediaPipe is applied on the RGB image obtained from the RealSense camera. By additionally including depth information, the person is located with respect to the robot. Then, their personal space is modeled with a Gaussian Mixture Model, where the space is larger toward the direction the person is looking at. This information is merged into the velocity map by applying the Haddamard product. In this way, when recalculating the path, the robot will not enter this area, being more user friendly than other traditional navigation methods (Mora et al., 2022b). A video of the proposed method is presented in https://youtu.be/qCg3jC__fO4 and a summary on how the navigation strategy is executed is shown in Figure 8.

#### 3.2.3 Manipulation learning

In the resolution of everyday tasks in the home, humans have developed a great ability to perform them regardless of their complexity. These skills have “tricks” that each user can have to perform a certain task in an optimal way in their environment. The generalization and programming of these processes is highly complex for a robot, as the tasks they face are different, the objects are manipulated in more or less complex ways and the environments in each home are adapted to the users.

In order for ADAM to be able to adapt to different types of tasks, objects and environments we have decided to make use of Imitation Learning (IL), also known as Learning from Demonstration (LfD). In broader terms, IL represents a method for acquiring and honing new skills by observing these skills being performed by another agent. In the context of robotics, IL serves as a technique to simplify the exploration of complex search spaces. When exposed to either successful or unsuccessful instances, it allows for a reduction in the search for potential solutions. This can be achieved by either commencing the search from a observed effective solution or, conversely, by eliminating what is recognized as an unsatisfactory solution from the search space. IL provides an inherent approach to train a robot, with the aim of reducing or even eliminating the need for explicit and laborious programming of a task by a human operator. As a result, these types of methods presents a “intuitive” way to program a robot, designed to be accessible to individuals without extensive technical expertise. The use of this type of learning has been applied to different tasks within assistive robotics for elderly people as can be seen in Joshi et al. (2019) where a work to help elderly people to get dressed is presented or in the work of Laskey et al. (2017) where an IL application is presented to make the bed in a robust way by means of camera-arm coordination.

The ADAM robot has therefore served as a testing ground for different Imitation Learning techniques and algorithms for solving tasks in the home environment. Firstly, we focus on solving the most commonly used manipulation tasks in the domestic environment: reaching, pushing and pressing. The combination of these sub-tasks allows us to generate more complex tasks such as placing objects on a table or the ability to manipulate household appliances for cooking tasks (e.g. putting a glass in a microwave and turning it on). For this process, a proprietary imitation algorithm, Fast Marching Learning (FML), was developed and presented in Prados et al. (2023b). This algorithm is based on the use of velocity caps generated by Fast Marching Square (FM^2^) which are modified by user demonstrations. An execution of this method is presented in https://youtu.be/_sklRg0NCM8. In addition to this, modifications have been made to this algorithm using its computational advantages such as the absence of local minima or that it is always able to return a possible solution. These modifications make use of the FML algorithm in conjunction with elastic maps, which allow better fitting of the data estimated by this algorithm, thus allowing constraints to be added to the tasks, such as additional grip points or objects to be taken into account that were not previously estimated in the generated demonstrations. The velocity maps generated through FML allow the paths learned by demonstrations to be probabilistically marked, thus encouraging the generation of optimal solutions. If we add to this the use of elastic maps, we generate not only an imitation learning algorithm that generates optimal paths, but that can be easily modified by means of spring meshes that “pull” the solutions to adapt them to certain critical points such as relevant points through which the solution has to pass (for example to grab objects that did not exist in the demonstration) or obstacles that have to be avoided. To evaluate the efficiency of the Elastic-FML method, tests have been performed using the LASA (Khansari-Zadeh and Billard, 2011) and RAIL (Rana et al., 2020) datasets which hav e reach, pull and push task demonstrations. The results (Table 4) show that the union of both methods generates better results in terms of Frechet's evaluation (which measures spatial dissimilarity), sum of squared error (SSE), which measures temporal and spatial dissimilarity and the angular distance which measures the difference between the demonstration and the result.

**Table 4:** Comparison of results from selected shapes from the LASA (2D) and RAIL (3D) datasets.

	**Name**	**Fréchet**			**SSE**			**Angular**		
		**Elastic map**	**FML**	**EFML**	**Elastic map**	**FML**	**EFML**	**Elastic map**	**FML**	**EFML**
2D	DoubleBendedLine	0.094	0.165	**0.090**	0.071	0.295	**0.070**	0.285	0.397	**0.271**
	Heee	**0.106**	1.000	0.347	**0.065**	0.471	1.000	**0.385**	0.934	1.000
	Rshape	**0.131**	0.173	**0.131**	**0.081**	0.105	0.083	0.492	0.626	**0.482**
	Spoon	**0.102**	0.133	**0.102**	0.057	0.099	**0.054**	0.480	0.464	**0.454**
	WShape	0.143	0.285	**0.132**	**0.172**	0.481	0.179	0.762	0.883	**0.705**
3D	Pushing	0.024	0.026	**0.019**	0.083	0.314	**0.071**	**0.533**	0.561	0.540
	Reaching	0.032	0.023	**0.021**	0.041	0.049	**0.038**	**0.553**	0.576	**0.553**
	Pressing	0.027	0.021	**0.017**	0.232	0.384	**0.147**	**0.557**	0.571	**0.557**

Bold numbers indicate which method obtained the best values for the proposed metrics.

In addition to these tests, a comparison of Elastic-FML with other LfD representations was also performed (Table 5). These representations include Correlated Dynamic Movement Primitives (CorrDMP), Gaussian Mixture Models with Mixture Regression (GMM/GMR), and Probabilistic Movement Primitives (ProMP).

**Table 5:** Comparison for the pick-and-place skill between EFML and several other LfD methods.

	**Frechet**	**SSE**	**Angular**	**Jerk**
EFML	**0.484**	0.113	0.852	**0.904**
CorrDMP	1.000	1.000	1.000	1.000
GMM/GMR	0.866	0.096	0.831	0.984
ProMP	0.991	**0.091**	**0.787**	0.992

Bold numbers indicate which method obtained the best values for the proposed metrics.

**Figure 9:**
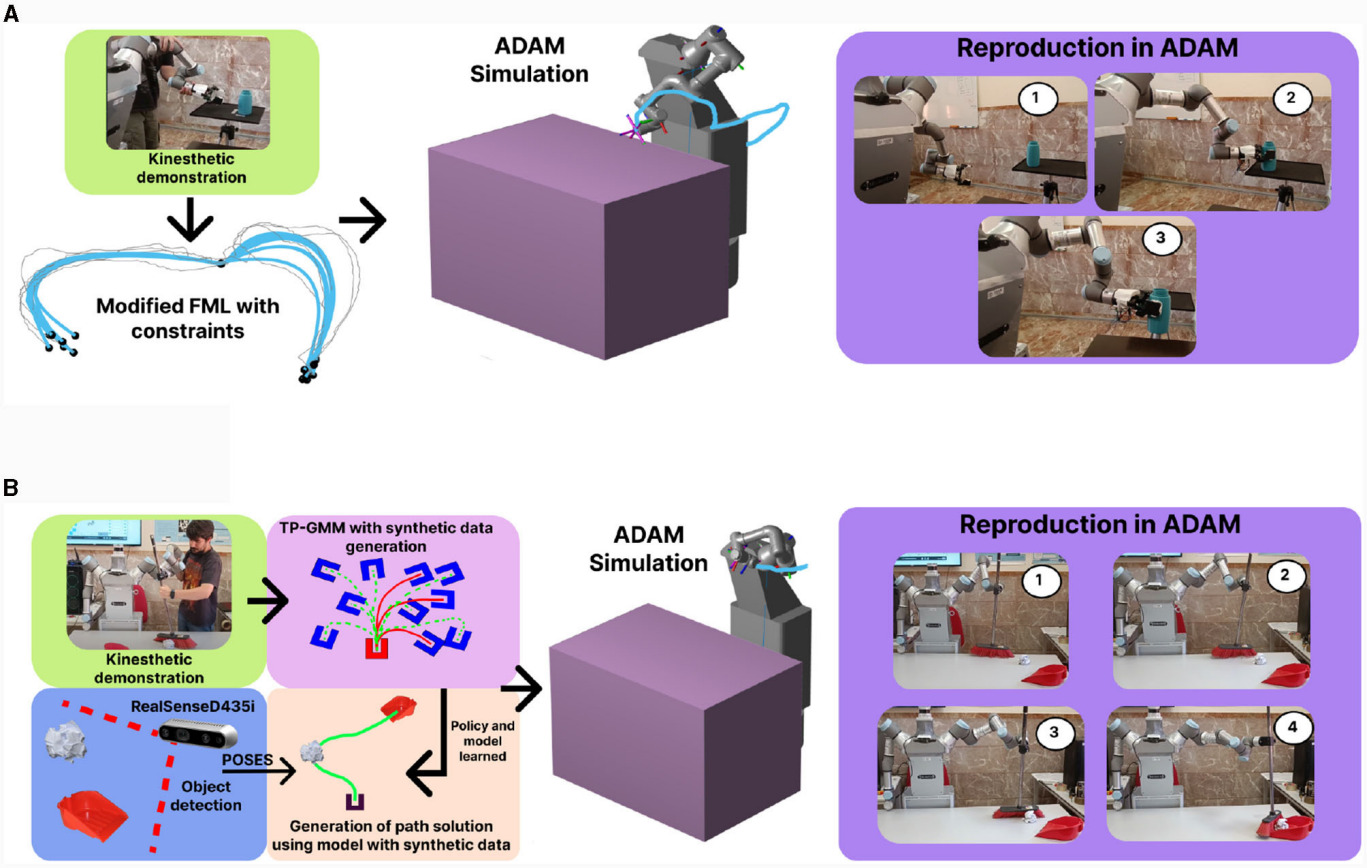
**(A)** Example of FML presented in Prados et al. (2023b). The FML algorithm allows the generation of the velocity field based on the demonstration data taken directly from the arms. The modification of FML by means of elastic maps additionally allows to take into account constraints such as grabbing a glass that we previously did not have in the scene. **(B)** Example of a sweep task using FML and TPGMM. In this case the generation of the demonstrations is done by moving the arm with the broom but new synthetic data is generated and compared with the human data in order to have a greater number of demonstrations for the application of parameterised tasks. This allows the sweeping task to be generalized to previously undemonstrated data.

This table reveals that EFML excels over alternative representations in specific categories, notably in Fréchet distance and jerk, with jerk representing the total jerk of the reproduction. Moreover, EFML demonstrates comparable performance to other methods in terms of SSE and angular distance metrics. Notably, the EFML reproduction exhibits lower jerk than its counterparts, signifying that it facilitates smoother and safer execution on real robots. This comparative analysis underscores EFML's ability to outperform other LfD algorithms. An example of the solution provided by this algorithm is presented in Figure 9A and is also presented in a video: https://youtu.be/TiMh-ilXh8g

The ADAM robot accomplishes another task, which is the act of sweeping. To achieve this, a combination of the FML algorithm and Task Parameterized Gaussian Mixture Models (TPGMM) has been employed (Calinon, 2016). The application of TPGMM allows us to estimate various initial and final orientations for the sweeping process. Generating new configurations for this task can be challenging, as TPGMM requires a substantial volume of data. Consequently, we have developed an algorithm to address this issue in the resolution of tasks, such as the sweeping task. The algorithm is based on a modification of the previously described FML algorithm, which we use to generate synthetic data solving sweeping tasks in a two-dimensional environment. These synthetic demonstrations must be at least as effective as those generated by humans. Therefore, through a cost function based on Wasserstein distance measurement (which allows us to quantify the disparity between human and synthetically generated data), we can selectively choose only those data points that are as good as human-generated ones. This enables us to generate a large amount of data with few human interactions, facilitating the creation of much more optimal learning models for the resolution of parameterized tasks, such as area sweeping. Figure 9B presents a brief example of that process. To assess the efficiency of this method compared to other methods that generate synthetic data, an environment has been established where the length of the generated solution (with shorter and more direct solutions considered better), the endpoint error (measuring how close it gets to the actual endpoint), and constraint satisfaction (evaluating collisions or impossible movements by the arm in task execution) have been measured. The developed algorithm has been compared against RF + Noise (Zhu et al., 2022) and αTP-GMR (Sena et al., 2019), and the results can be observed in Table 6.

**Table 6:** Comparison between the three algorithms for different initial and final frames.

	**Trajectory lengths**		**Trajectory end-points errors**		**Constrains satisfaction errors**	
	**Mean (m)**	**Std**.	**Mean (m)**	**Std**.	**Mean (m)**	**Std**.
RF + Noise	3.98	±0.87	0.080	±0.00	18.75	±2.81
αTP-GMR	5.73	±1.40	0.040	±0.00	**1.00**	±0.00
**Our method**	**2.29**	±0.53	**0.038**	±0.00	**1.00**	±0.00

Bold numbers indicate which method obtained the best values for the proposed metrics.

The RF + Noise algorithm exhibits significant constraint satisfaction errors, particularly when dealing with orientations different from those in the demonstrations, due to collisions. In contrast, both the αTP-GMR algorithm and the one introduced in this paper do not encounter this issue. Both algorithms produce valid and closely aligned solutions. However, when evaluating results based on path length, our algorithm outperforms the αTP-GMR algorithm. Specifically, the average length of the solution paths generated by our algorithm is considerably shorter than those produced by the αTP-GMR algorithm, indicating that our approach yields more optimal results in terms of path length. An example of that algorithm is presented in this video: https://youtu.be/pD1HdoWJmfs.

**Figure 10:**
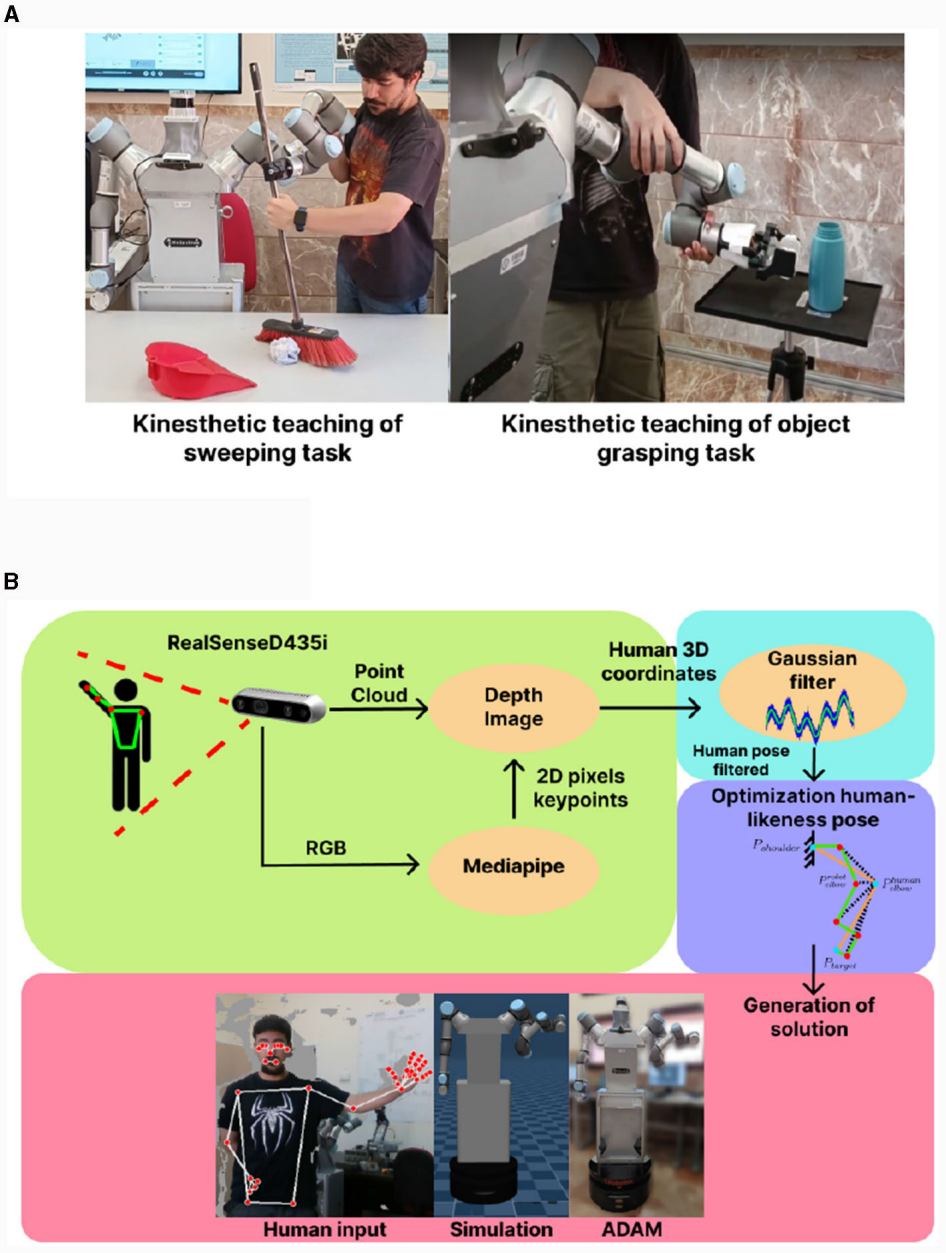
**(A)** Example of kinesthetic data acquisition for sweeping and grasping tasks. The arm works in gravity compensation so it is easy to move it and record data directly from the arm itself. **(B)** Example of the logic used by the TAICHI algorithm for user data collection by RGBD camera presented in Lopez et al. (2023) and Prados et al. (2023a). The algorithm detects the user and his hands, tracks and filters the noise and then optimizes the human configuration to the non-anthropomorphic robotic arm by means of the POSE of the shoulder, elbow and wrist.

Another relevant and very important factor in the imitation learning process pertains to data acquisition. To facilitate user engagement with these data, we have devised two distinct approaches for this purpose. The first approach involves kinesthetic data acquisition (presented in Figure 10A), wherein the robot's own arm is employed in a gravity compensation mode, enabling control by the operators. This approach empowers the user to directly consider the inherent limitations of the robot's arm while performing tasks. Data is collected as the user manipulates the arm for the required task and subsequently subjected to filtering to eliminate potential redundancies. Despite its effectiveness there may be users who have limitations in moving the arm, therefore we have developed a mimicry algorithm that, by using the RGB-D camera of the ADAM robot, generates movement data for the arms taking into account the orientation and position of the relevant points of the arm (shoulder-elbow-wrist). The created algorithm Tracking Algorithm for Imitation of Complex Human Inputs (TAICHI) presented in Lopez et al. (2023) and Prados et al. (2023a) allows the generation of arm movement data that is safe with itself and with the environment, easy to take by the user and without the need to have the robot active for it, as it makes use of simulations to check its effectiveness. A graphical explanation of the method is presented in Figure 10B. The TAICHI algorithm begins with a user detection process using the RealSense camera (presented in Section 3.1.1), and utilizes MediaPipe (presented in Section 3.2.1) to estimate the person's skeleton. A Gaussian filter is applied to reduce noise in the camera data. The algorithm focuses on characteristic points of the human arm (shoulder-elbow-wrist). As the UR3 arm used in the robot ADAM is non-anthropomorphic, an approximation is made using a cost function. This function evaluates the distance between the robot's elbow and the human's elbow, seeking to minimize this distance. It takes into account relevant factors such as wrist orientation, movement continuity, and the absence of collisions with both itself and the surrounding objects. The algorithm thus converts human configurations for a task into a series of ADAM robot configurations, directly adapting them to its characteristics. The algorithm has been tested in both simulation and real robot movements, as demonstrated in the following video: https://youtu.be/rSynqgXa_Yc. To assess the efficiency of the TAICHI algorithm, a validation was conducted based on the error in position and orientation obtained for various demonstrations performed by individuals of different heights and physical constitutions (Table 7). As observed, the results indicate that the error in position and orientation remains consistently low, demonstrating that the algorithm can effectively generalize from any human arm to the ADAM robot arm.

**Table 7:** Position and orientation errors of TAICHI algorithm for different tests.

	**Max outlier error**				**Mean error**			
	*x* **(m)**	*y* **(m)**	*z* **(m)**	Θ **(rad)**	*x* **(m)**	*y* **(m)**	**z (m)**	Θ **(rad)**
Test 1	0.0230	0.0310	0.0241	0.1530	0.0014	0.0018	0.0014	0.0097
Test 2	0.0233	0.0311	**0.0207**	0.1147	0.0024	0.0022	0.0017	0.0084
Test 3	0.0294	0.0177	0.0156	0.0551	0.0050	0.0028	0.0027	0.0062
Test 4	0.0330	**0.0401**	0.0274	**0.2011**	**0.0100**	**0.0052**	**0.0610**	**0.0146**
Test 5	**0.0350**	0.0300	0.0170	0.1316	0.0036	0.0031	0.0030	0.0175
Test 6	0.0260	0.0160	0.0190	0.1543	0.0019	0.0021	0.0032	0.0187

Bold numbers indicate which method obtained the best values for the proposed metrics.

#### 3.2.4 Communication between modules and other robotic platforms

**Figure 11:**
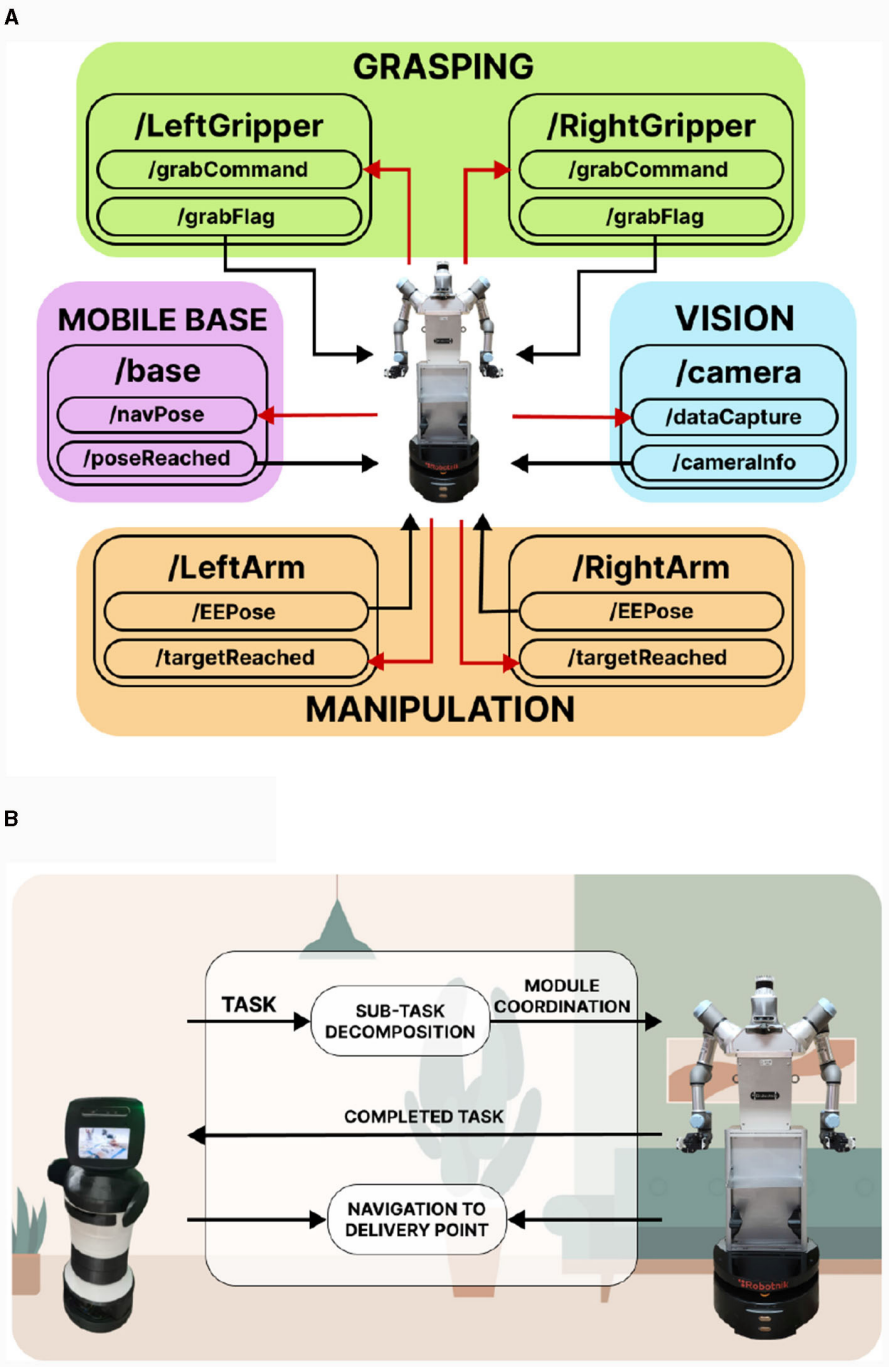
ADAM robot communications: **(A)** Communication schematic between the robot modules. A total of six modules make up the operation of the robot: two for the grippers, two for the arms, one for the mobile base and one for vision. The red arrows indicate the information sent from the controller to the modules and the black arrows from the modules to the controller. **(B)** Communications with another robot. ADAM receives a task, which is decomposed into subtasks assigned to each of its modules. Once executed, ADAM communicates with the other robot to indicate that it has finished and establishes a meeting point in case it needs to exchange objects.

Once the modules that make up the ADAM robot have been presented, it is important to highlight the need for all of them to be properly communicated with each other in order to be able to perform complex tasks. The environment in which the described modules have been programmed is ROS, a widely used middleware in the robotics world that provides the necessary tools for different robotic elements to communicate with each other. Each of the different modules and algorithms developed on the robot ADAM are independent and use the ROS system solely for module-to-module communication. Therefore, each algorithm takes into account specific safety criteria for each type of task and communicates it through topics to the rest of the modules when necessary. This communication capability allows, for example, the vision module to inform the manipulation module through ROS about the location of dirt during a sweeping task, enabling the manipulation module to act on that element and communicate arm position and orientation continuously. This ensures that, despite being modular, the system is always communicating, and the user can be aware of the actions and values each module is taking. Depending on the task ADAM is to perform, the robot modules must operate in a specific order. For this purpose, a module sequencer is used to switch between modules at the appropriate time. The scheme of the proposed communications is shown in Figure 11A. As an example, the task of bringing a bottle to the user is used. First, the robot must navigate to the kitchen, so the “MOBILE BASE” module is activated. By using maps, ADAM is able to navigate to the room labeled as “kitchen.” Once there, the “VISION” module is in charge of detecting the object and returning its pose information so that the “MANIPULATION” module can then move the robotic arms to the corresponding point. The “GRASPING” module is activated to grab the object and finally the “MOBILE BASE module is activated again to return to the place where the user is to deliver the bottle.

Thanks to the communication features of ROS, our robot is also able to work together with other platforms. Since ADAM is a physical assistive robot, it can work in coordination with cognitive robots to facilitate communication with the user. A scheme of the proposed architecture can be seen in Figure 11B. First, ADAM can receive a command indicating the task to be performed from another robot, such as the previously described task. The task is decomposed into the sequence of modules that must be activated and deactivated in order to perform it. Once this task is performed, ADAM sends a signal indicating that the task is complete. Finally, in the case that the task involves the need to transport an object to the user, the robots go to a delivery point where the object is exchanged.

## 4 Applications in real environments

The ADAM robot has been tested in various applications in everyday life and in projects involving users. The tasks it has demonstrated proficiency in within the everyday context include object sweeping, object rearrangement, and the preparation of simple meals, delivering them to the user by navigating safely through the environment. This section presents two specific examples of such tasks. Firstly, a specific daily task involving the arrangement of necessary utensils for eating is described, showcasing the use of all the modules and communication between them as presented in this work. Secondly, a collaborative project with the University of Cartagena (Spain) and the University of '´Orebro (Sweden) is outlined. This project introduces a multi-robot system in an assisted home environment to support elderly individuals in their daily lives. As a specific example, the project aimed at having the ADAM robot prepare a simple meal and deliver it to the user's position.

### 4.1 ADAM the waiter: table setting task

Once they are operational, the social navigation, vision and manipulation modules are tested jointly to complete a real home task which consists on setting the table. The environment of this task consists of a room with two tables 4 m apart from each other. Table A where the objects (a cup and a water bottle) are initially located, and table B as the one to be set.

For this purpose, it is created a master node which centralizes the flow of information between the individual modules and controls the execution sequence of 4 different actions (navigation, object grasp, object drop and water pour) which are associated to robot's modules, as detailed in Section 3.2.4. In navigation, the robot executes the FM^2^ algorithm to displace between tables A and B avoiding collisions with any room objects detected using the laser sensor on its base. For the object grasp action, the robot identifies the object to be grasped and obtains the grasp point which is later sent to the FML algorithm as the target point for the end effector on the robotic arm. When the arm reaches the target position, a signal is sent the gripper to close its jaws and grab the object. To drop the object, the gripper opens and the robot arm returns to rest initial position. The water pour action begins by identifying a cup and obtaining its location which is later sent to the manipulation algorithm to move the robot to the target position above the cup and rotate the end effector to pour the water.

**Figure 12:**
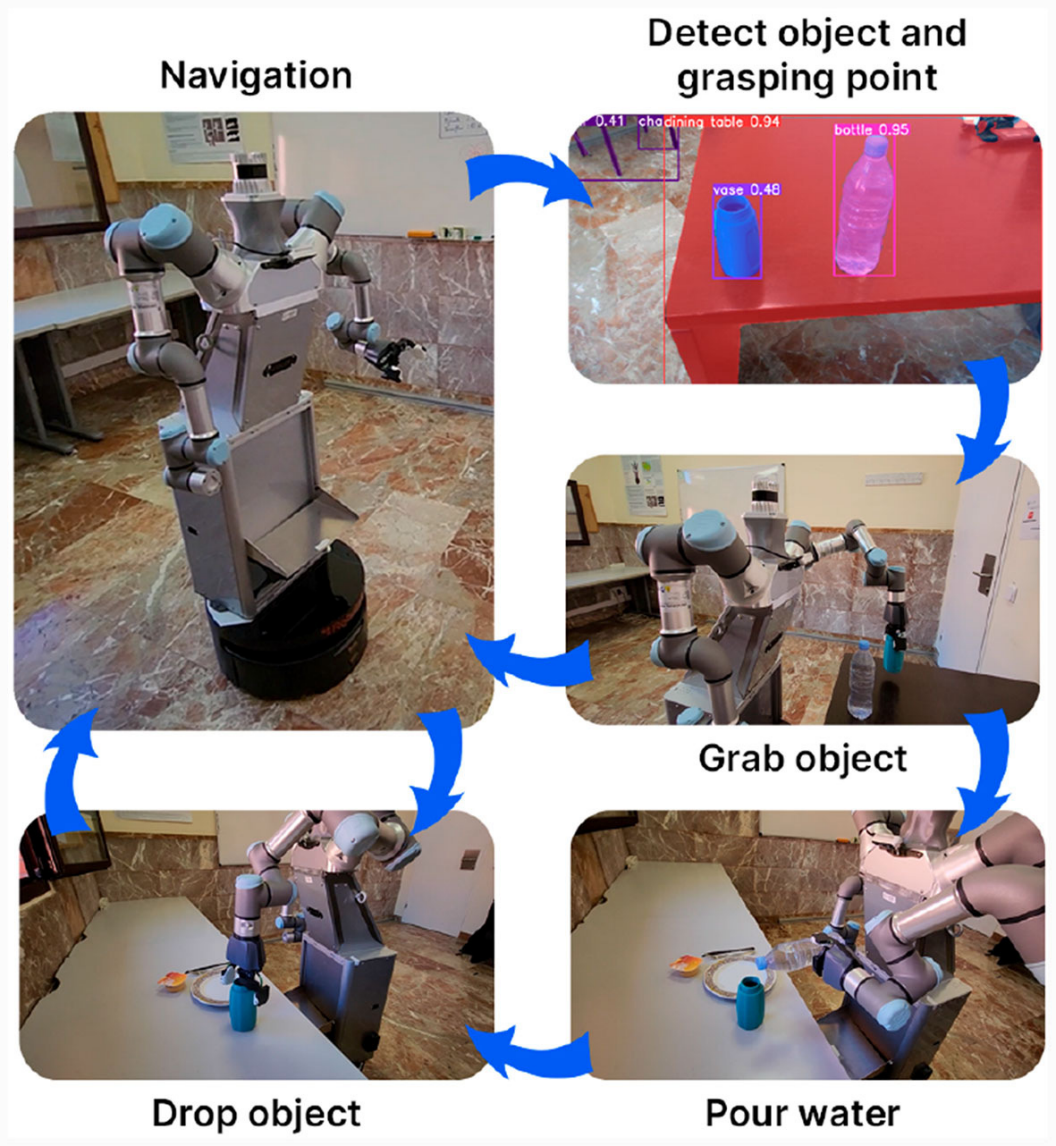
Joint operation of the functioning modules of the ADAM robot to set the table as a complete home task. The robot performs four different actions (navigation, object grasp, object drop, water pour) initiated by the master node which communicates the different robot modules.

These four actions are combined as shown in Figure 12 to complete the task. The routine starts with the robot in its initial position and moving toward table A where the cup and the bottle are placed. The robot then identifies and grabs the cup and once the cup is held secure, the robot raises its arm and moves toward table B. The cup is then placed in table B next to the cutlery and returns to table A to grab the next object. Again, the robot identifies the grasping point of the bottle and closes the gripper to grab it. The robot carries the bottle to table B and identifies the position of the cup previously placed moving the arm with the bottle above it and turning the gripper to pour the water. Lastly, the robot places the bottle on table B and displaces to the initial position to end the task. Thus, this experiment is considered as successful when the base arrives correctly to the pick-up position and the final position, objects are held secure without being damaged during all trajectories, when the objects are placed at the correct locations and the robot modules send the corresponding messages to achieve a correct and fluent communication. In order to obtain the success rate of the table setting task, we have carried out 6 experiments with different positions and orientations of objects as well as objects of different weights and in different rooms. In all cases, the algorithm was able to successfully complete the manipulation part, reaching the required position of each object, grasping the objects and not letting them stray, and the navigation part, reaching both the pick-up point and the positioning point of the objects.

An example of one of this experiments is presented in https://youtu.be/9KxwCN91rDA?si=B96lY7wxvRw2ppb2. This video demonstrate the correct individual operation of the vision, navigation and manipulation algorithms implemented, as well as the fluent and efficient communication between them to complete the table setting task.

### 4.2 HIMTAE project

**Figure 13:**
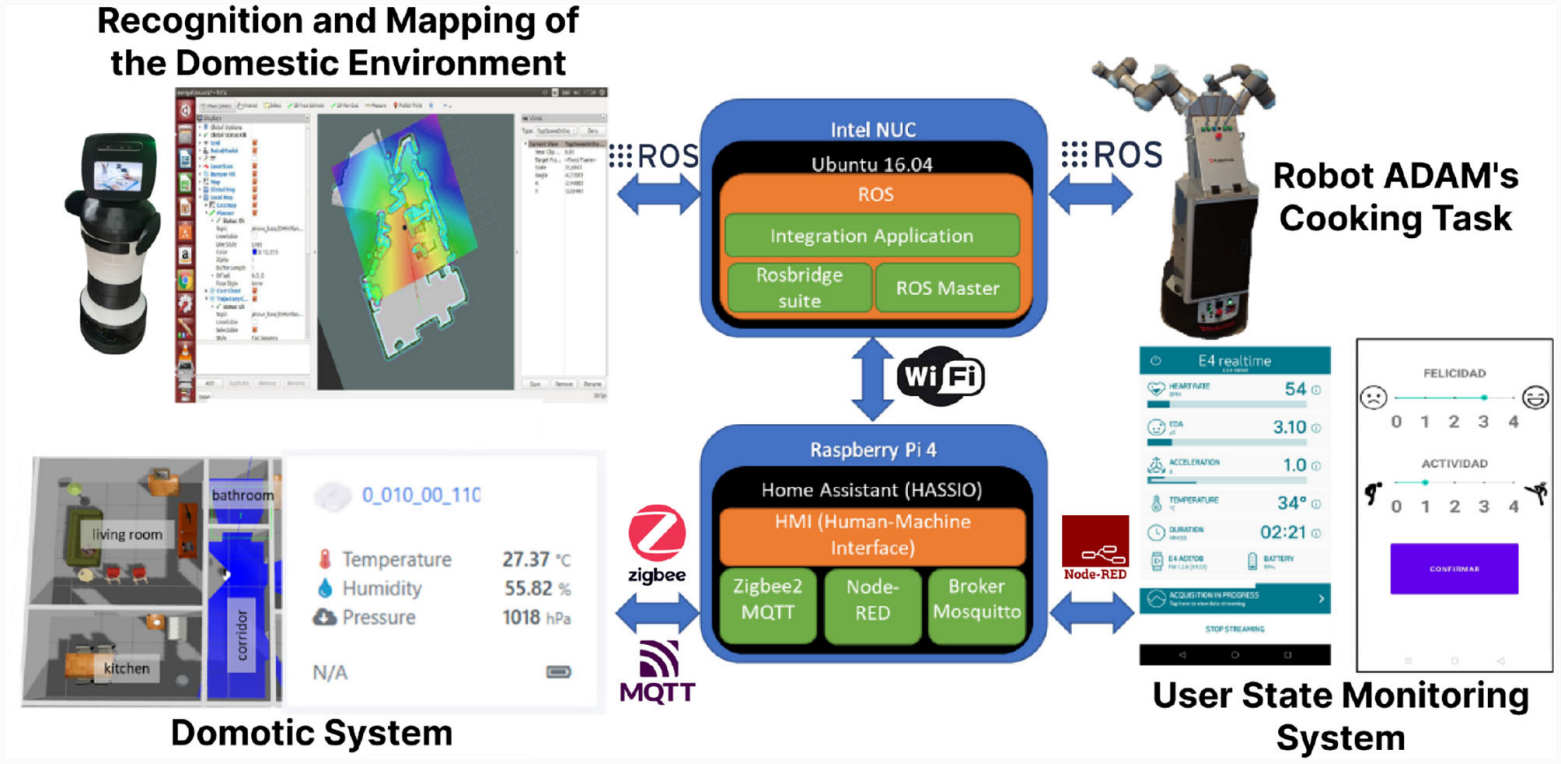
General integration scheme of the multirobot system with the home automation system and user control bracelet presented in Barber et al. (2022b,a). The system is mounted on two different hardware platforms. The user data analysis and home automation control components are implemented on a Raspberry Pi 4, using communication via MQTT, Zigbee, and Node Red to exchange information and activate system elements. Communication with the Intel NUC, which serves as the master ROS node, is established through the home's own WiFi network. This hardware is responsible for facilitating communication between the two robots, handling user input such as requesting specific tasks in the kitchen, as well as sharing sensor data, including environment maps necessary for the real-time location of each robot and the user.

The Heterogeneous Intelligent Multi-Robot Team for Assistance of Elderly People (HIMTAE) project (Barber et al., 2022a,b) was a collaboration with the University of Cartagena in Spain and the the University of '´Orebro in Sweden, where the aim was to present an application for the care of elderly people in an Ambient Assisted Living (AAL) by monitoring and assisting them in the tasks they require. The main idea was to prove that the ADAM system developed as well as the assistance and home automation systems presented could be useful with real users in their homes. For this purpose, the project was based on three different parts explained below and presented visually in Figure 13:

- Domotic system: the homes of the elderly were adapted with domotic systems that allowed them to control the temperature, lights or blinds of the home.
- Assistive robotics for the elderly: a robot was created through the Robwell project which was responsible for establishing an empathetic relationship with the elderly user as well as serving as a node to send orders to both the home automation system and the ADAM robot to carry out physical tasks. Additionally, users had a bracelet that monitored their physical and mental states and were communicated to Robwell, which was in charge of communicating or reminding them to drink water according to their specific needs.
- Assistive robot for physical tasks: this part was carried out by the ADAM robot as part of the HEROITEA (Heterogeneous Social-Mobile Manipulator Robot Intelligent Teams for Elderly-People Assistance) project. The robot was constantly in communication with the Robwell robot which, depending on the user's needs, sent them to ADAM so that it could carry them out. In this project ADAM was limited to solving tasks in the kitchen, focusing on preparing dishes for the users. Once it was ready, ADAM took the food to Rowell who was responsible for giving it to the users. This video present the kitchen task of the ADAM robot: https://youtu.be/iV0hNSYEhVM.

In this project, satisfactory results were presented in the different fields of study. Firstly, the elderly people who used the multi-robot system were highly satisfied with the domotic systems, the interaction with the assistive robot and with the ADAM robot that helped them to prepare certain food in the kitchen. Secondly, it could be observed that the use of a multi-robot system was highly efficient because while the assistive robot was constantly in the human environment and interacting with the user, the ADAM robot could be able to perform the task in parallel. This made it possible to avoid situations of frustration on the part of the user if the task was delayed. Finally, we were able to directly interact the ADAM robot with real users, as the users could enter the kitchen while the robot was performing a task, and the ADAM robot could be able to interact with the user while the user was performing a task. Although the users tested were highly satisfied with the tasks performed by the robots (with an average value of 93% satisfaction), these results have been taken as tests in controlled laboratory environments and therefore cannot be extrapolated to real domestic environments, where future tests will seek to verify real satisfaction.

## 5 Conclusions and future work

This article introduces the ADAM robot, a modular mobile manipulator robot to provide physical assistance to elderly people at home. We have shown that ADAM is able to perform everyday household tasks such as setting the table or cleaning the floor that can be tedious or complicated for elderly people. Furthermore, ADAM has the ability to cooperate with other robotic platforms by communicating with them for tasks where more than one robot is needed. In addition to this, ADAM has been presented not only as an elderly care platform, but also as a platform for the development of new algorithms within the RoboticsLab.

With the current developments, ADAM has reached a certain degree of maturity in various capabilities such as human awareness, learning from the user, detection and recognition of the environment and navigation and comprehension of complex scenarios. These capabilities allow the robot to provide elderly people with the assistance they need by working safely, accurately and adapting to them and the environment. However, there are some issues that can be an impediment to provide a complete assistance to elderly people. The perception system is fixed so in certain situations ADAM will not be able to detect specific parts of the environment. The bimanipulation capabilities of ADAM are not fully developed and the arms configuration is not optimized. Therefore, it is essential to solve this issues to improve the assistance that the robot offers to elderly people.

Future lines of work on the ADAM robot will be related to hardware improvements and expansions and the development of new software that allows to better exploit the robot's vision, manipulation and navigation modules. This includes the implementation of a robotic neck to support a head containing the vision sensors, allowing to automatically adjust the robot's point of view, a redesign of the arms to expand its workspace and the integration of robotic hands to be used in grasping applications where more precision is required. In order to take advantage of the capabilities of the robot, future research will also focus on the use of 3D information to generate new environment representations for safer manipulation and navigation considering a complete model of the robot and its real time configuration. Lastly, new task and motion planning strategies will be implemented to deal with more complex home tasks, which will make ADAM a much more complete robot companion for elderly care.

## Data availability statement

The datasets generated for object localization using point cloud can be found in Kaggle: https://www.kaggle.com/datasets/aliciamorav/object-segmentation-dataset. All the codes presented in this papers can be found in the next repositories. The FML algorithm is fully explained and presented in https://github.com/AdrianPrados/FastMarchingLearning. The TAICHI code can be found fully explained and presented in https://github.com/AdrianPrados/TAICHI. The simulator for ADAM is presented in https://github.com/vistormu/adam_simulator.

## Author contributions

AMo: Conceptualization, Data curation, Formal analysis, Investigation, Methodology, Resources, Software, Validation, Visualization, Writing – original draft, Writing – review & editing. AP: Writing – review & editing, Conceptualization, Data curation, Formal analysis, Investigation, Methodology, Resources, Software, Validation, Visualization, Writing – original draft. AMe: Conceptualization, Data curation, Formal analysis, Investigation, Methodology, Resources, Software, Validation, Visualization, Writing – original draft, Writing – review & editing. GE: Conceptualization, Data curation, Formal analysis, Investigation, Methodology, Resources, Software, Validation, Visualization, Writing – original draft, Writing – review & editing. PG: Formal analysis, Software, Visualization, Writing – review & editing. BL: Software, Writing – review & editing. VM: Software, Writing – review & editing. LM: Conceptualization, Project administration, Supervision, Writing – review & editing. SG: Conceptualization, Funding acquisition, Project administration, Supervision, Writing – review & editing. RB: Conceptualization, Funding acquisition, Project administration, Supervision, Writing – review & editing.
